# Synergistic immunotherapy of glioblastoma by dual targeting of IL-6 and CD40

**DOI:** 10.1038/s41467-021-23832-3

**Published:** 2021-06-08

**Authors:** Fan Yang, Zhenqiang He, Hao Duan, Duo Zhang, Juehui Li, Huijuan Yang, Jay F. Dorsey, Wei Zou, S. Ali Nabavizadeh, Stephen J. Bagley, Kalil Abdullah, Steven Brem, Lin Zhang, Xiaowei Xu, Katelyn T. Byrne, Robert H. Vonderheide, Yanqing Gong, Yi Fan

**Affiliations:** 1 Department of Radiation Oncology, University of Pennsylvania, Philadelphia, PA USA; 2 Department of Radiology, University of Pennsylvania, Philadelphia, PA USA; 3 Abramson Cancer Center, University of Pennsylvania, Philadelphia, PA USA; 4 Department of Neurosurgery, University of Pennsylvania, Philadelphia, PA USA; 5 Department of Obstetrics and Gynecology, University of Pennsylvania, Philadelphia, PA USA; 6 Department of Pathology and Laboratory Medicine, University of Pennsylvania, Philadelphia, PA USA; 7 Institute for Immunology, University of Pennsylvania, Philadelphia, PA USA; 8 Division of Human Genetics and Translational Medicine, Department of Medicine, University of Pennsylvania, Philadelphia, PA USA; 9 Present Address: State Key Laboratory of Oncology in South China, Department of Neurosurgery/Neuro-oncology, Sun Yat-sen University Cancer Center, Collaborative Innovation Center for Cancer Medicine, Guangzhou, China

**Keywords:** CNS cancer, Cancer immunotherapy

## Abstract

Immunologically-cold tumors including glioblastoma (GBM) are refractory to checkpoint blockade therapy, largely due to extensive infiltration of immunosuppressive macrophages (Mϕs). Consistent with a pro-tumor role of IL-6 in alternative Mϕs polarization, we here show that targeting IL-6 by genetic ablation or pharmacological inhibition moderately improves T-cell infiltration into GBM and enhances mouse survival; however, IL-6 inhibition does not synergize PD-1 and CTLA-4 checkpoint blockade. Interestingly, anti-IL-6 therapy reduces CD40 expression in GBM-associated Mϕs. We identify a Stat3/HIF-1α-mediated axis, through which IL-6 executes an anti-tumor role to induce CD40 expression in Mϕs. Combination of IL-6 inhibition with CD40 stimulation reverses Mϕ-mediated tumor immunosuppression, sensitizes tumors to checkpoint blockade, and extends animal survival in two syngeneic GBM models, particularly inducing complete regression of GL261 tumors after checkpoint blockade. Thus, antibody cocktail-based immunotherapy that combines checkpoint blockade with dual-targeting of IL-6 and CD40 may offer exciting opportunities for GBM and other solid tumors.

## Abstract (web summary)

Glioblastomas are generally resistant to treatment with immune checkpoint inhibitors. Here the authors show that IL6 blockade, in combination with a CD40 agonist, overcomes macrophage-mediated immunosuppression and sensitizes glioblastoma to immune checkpoint blockade in preclinical models.

## Introduction

Immunotherapy holds great promise for cancer treatment. However, current immunotherapy approaches against solid tumors remain a significant challenge, in particular for immunologically cold tumors, i.e., those characterized with low T-cell infiltrates, including glioblastoma (GBM)^1–6^. In these tumors, the therapeutic difficulties and failures are largely due to an immune-hostile, suppressive tumor microenvironment that abrogates T-cell infiltration and activation. GBM, the grade IV glioma, is the most common primary malignant brain tumor in adults. GBM is among the most lethal of human malignancies, with a median survival of around 14–16 months. GBM is highly resistant to standard therapies, including surgical resection, radiation, and chemotherapy^7,8^. Consistent with its immunologically cold nature resulted from an extraordinary immunosuppressive microenvironment, GBM is generally refractory to T-cell-based immunotherapies including PD-1/PD-L1-targeting checkpoint inhibition and adoptive cell transfer with chimeric antigen receptor-modified T cells^2,9–11^. Development of effective strategies for reversal of tumor immune suppression is, therefore, crucial for a successful immunotherapy against GBM.

Tumor-associated macrophages (Mϕs) play a pivotal role in tumor progression, cancer immunosuppression, and therapy resistance^12–14^. A prominent population of Mϕs in the tumor microenvironment executes tumor-promoting functions: secreting growth factors and releasing immunosuppressive cytokines, such as interleukin-10 (IL-10), transforming growth factor-β (TGF-β), and arginase-1, at least partially via alternative Mϕ polarization^15–18^. Notably, Mϕs are a major population of the non-neoplastic cells in GBM, making up as much as half of the cells in GBM tumors^19,20^, suggesting tumor Mϕs as a major source for GBM immunosuppression. However, the precise mechanisms controlling Mϕs-mediated GBM immunosuppression remains largely unknown, and the better understanding of these mechanisms will help identify key therapeutic targets to activate anti-tumor immunity.

Our previous work shows that vascular niche-derived IL-6 induces alternative Mϕ activation in GBM, suggesting IL-6 as a therapeutic target for GBM immunotherapy^21^. Here we report that genetic ablation or pharmacological inhibition of IL-6 partially reverses Mϕ-mediated GBM immunosuppression but does not sensitize GBM to anti-PD-1/CTLA-4 treatment. Based on our transcriptome analysis that identifies an IL-6-inducible mechanism for Mϕ activation via Stat3/HIF-1α/CD40, we develop a dual-targeting anti-IL-6 and pro-CD40 strategy, which may offer exciting opportunities for activating Mϕ immunity and improving T-cell-based immunotherapy in solid tumors.

## Results

### IL-6 is critical for tumor immunosuppression in GBM

**Fig. 1:**
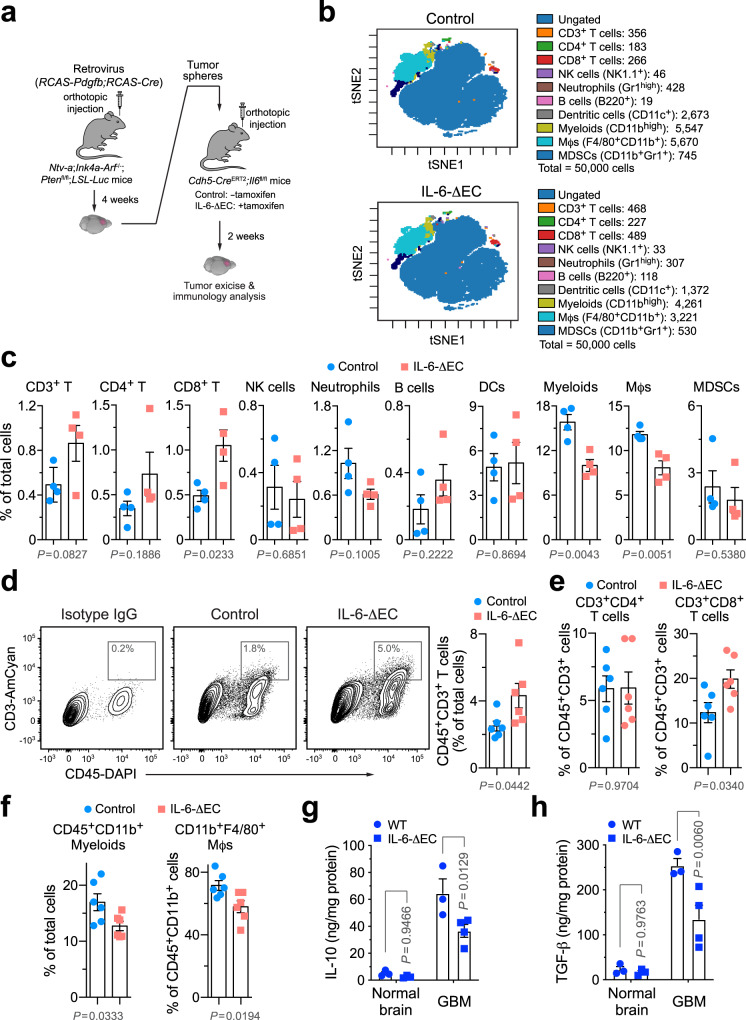
Genetic ablation of IL-6 reverses GBM immunosuppression. GBM was induced by RCAS-mediated genetic engineering in *Ntv-a*;*Ink4a-Arf*^−/−^;*Pten*^fl/fl^;*LSL-Luc* donor mice, followed by orthotopic tumor implantation into *Cdh5-Cre*^ERT2^;*Il6*^fl/fl^ recipient mice that were pretreated with (IL-6-ΔEC) or without (Control) tamoxifen. Two weeks after tumor implantation, tumors were excised. **a** Schematic approach. **b**, **c** Tumor-derived single-cell suspensions were analyzed by CyTOF. **b** Representative CyTOF sorting. **c** Quantitative results (mean ± SEM, *n* = 4 mice). Statistical analysis by two-tailed Student’s *t*-test. **d**–**f** Tumor-derived single-cell suspensions were analyzed by flow cytometry. **d** Analysis for CD3^+^ T cells. Left, representative cell sortings. Right, quantified results (*n* = 6 mice, mean ± SEM). Statistical analysis by two-tailed Student’s *t*-test. **e**, **f** Analysis for **e** CD4^+^/CD8^+^ T cells or **f** myeloid cells (*n* = 6 mice, mean ± SEM). Statistical analysis by two-tailed Student’s *t*-test. **g**, **h** Tissue lysates from normal brains and tumors were subjected to ELISA analysis for **g** IL-10 and **h** TGF-β expression (mean ± SEM, *n* = 4 mice for IL-6-ΔEC GBM group and *n* = 3 mice for other groups). Statistical analysis by two-way ANOVA with Sidak’s test. Source data are provided as a Source data file.

We investigated the role of IL-6 for tumor immunosuppression in GBM, initially using a genetic approach (Fig. 1a). Considering tumor-associated endothelial cells (ECs) as a major source for IL-6 expression in GBM^21^, we utilized a tamoxifen-inducible, EC-specific gene-knockout system to precisely regulate IL-6 expression in the tumor microenvironment. GBM was induced in *Ntv-a*;*Ink4a-Arf*^−/−^;*Pten*^fl/fl^;*LSL-*Luc donor mice by RCAS (replication-competent avian sarcoma-leukosis virus long terminal repeat with splice acceptor)-mediated gene transfer, followed by orthotopic implantation of tumor cells into *Cdh5-Cre*^ERT2^;*Il6*^fl/fl^ mice, in which IL-6 expression is controlled by EC-specific promoter Cdh5. Mass cytometry (cytometry by time of flight, CyTOF) analysis of tumor-derived single-cell suspension showed that genetic ablation of IL-6 increased the population of cytotoxic CD8^+^ T cells in GBM (Fig. 1b, c). Moreover, similar increasing trends were observed in CD3^+^ and CD4^+^ T cells as well, whereas the population of natural killer (NK) cells remained unchanged. Notably, IL-6 knockout reduced the populations of total myeloid cells and Mϕs, suggesting that the increased T-cell recruitment or activation may be due to reduced infiltration of immunosuppressive Mϕs into the tumors. Consistent with these findings, flow cytometry analysis of tumor-derived cells showed that IL-6 knockout enhanced infiltration of CD3^+^ T cells into the tumors (Fig. 1d) with an increased portion of CD8^+^ T cells in CD45^+^CD3^+^ T cells (Fig. 1e and Supplementary Fig. 1), associated with reduced CD45^+^CD11b^+^ myeloid cells and CD45^+^CD11b^+^F4/80^+^ Mϕs (Fig. 1f). Tumor-associated Mϕs secrete a plethora of anti-inflammatory, immunosuppressive cytokines, such as IL-10 and TGF-β, which inhibit T-cell infiltration into and activation in the tumors. Our enzyme-linked immunosorbent assay (ELISA) analysis revealed that IL-6 ablation substantially reduced IL-10 and TGF-β expression in GBM tumors but not in normal brains (Fig. 1g, h). These findings collectively suggest a critical role IL-6 plays in GBM immunosuppression, supportive of our previous work showing that IL-6 knockout in ECs inhibits GBM growth and improves survival in tumor-bearing mice^21^. Furthermore, these results implicate that IL-6 blockade may represent a strategy to activate T-cell-based anti-tumor immunity.

### IL-6 neutralization improves animal survival but does not sensitizes GBM to immune checkpoint blockade

**Fig. 2:**
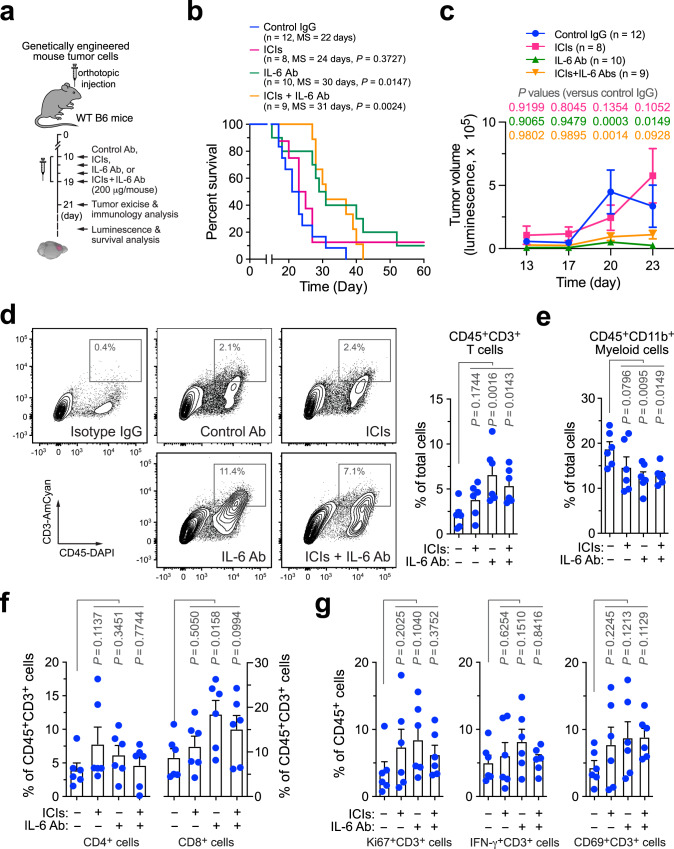
IL-6 neutralization enhances T-cell infiltration into GBM tumors and improves animal survival but does not sensitize tumor to immune checkpoint blockade. GBM was induced in WT B6 mice, followed by injection with control IgG, anti-IL-6 antibody (Ab), immune checkpoint inhibitors (ICIs), or ICIs plus anti-IL-6 Ab. **a** Schematic approach. **b**, **c** Survival and tumor growth analyses (*n* = 8–12 mice, specific *n* numbers are shown in the figure). **b** Mouse survival was monitored for 60 days and subjected to two-sided log-rank Mantel–Cox analysis. MS, median survival. **c** Tumor volume was analyzed by bioluminescence imaging during days 13–23 (mean ± SEM). Statistical analysis by two-way ANOVA with Dunnett’s test. **d**–**g** Tumors were excised 2 days after treatment. Tumor-derived single-cell suspensions were stained with antibodies against CD45, **d** CD3, **e** CD11b, **f** CD4, CD8, CD3, and **g** Ki67, IFN-γ, and CD69, followed by flow cytometry analyses. **d** Analysis for CD3^+^ T cells. Left, representative cell sortings. Right, quantified results (*n* = 6 mice, mean ± SEM). Statistical analysis by one-way ANOVA with Fisher’s LSD test. **e**–**g** Quantified results for immune cells (*n* = 6 mice, mean ± SEM). Statistical analysis by one-way ANOVA with Fisher’s LSD test. Source data are provided as a Source data file.

GBM is insensitive to immune checkpoint blockade due to low T-cell infiltrates in the tumors, which remains a significant challenge in the clinic^4^. Considering the negative role of IL-6 for T-cell infiltration and activation in the tumors, we next tested the therapeutic potential of a IL-6-neutralizing antibody, particularly in combination with immune checkpoint inhibitors (ICIs, anti-PD-1 plus anti-CTLA-4 antibodies) in the genetic mouse GBM model (Fig. 2a). Survival analysis showed that anti-IL-6 treatment moderately but significantly (*P* < 0.05) improved the survival by over 30% (+8 days, comparable to 22 days of median survival in control mice, Fig. 2b). Moreover, anti-IL-6 treatment inhibited tumor growth (Fig. 2c). In accordance with our results by genetic IL-6 ablation (Fig. 1), flow cytometry analysis showed that IL-6 neutralization enhanced infiltration of CD45^+^CD3^+^ T cells into the tumors (Fig. 2d) and reduced recruitment of CD45^+^CD11b^+^ myeloid cells and CD45^+^CD11b^+^F4/80^+^ Mϕs (Fig. 2e and Supplementary Fig. 2), but did not affect the populations of CD45^+^CD11b^+^Ly6G^High^Ly6C^Int^ neutrophils and CD45^+^CD11b^−^CD3^−^NK1.1^+^ NK cells (Supplementary Fig. 2). However, IL-6 neutralization moderately enhanced the ratios of CD8^+^ T cells in tumor-associated CD45^+^CD3^+^ T cells (Fig. 2f), but did not activate these T cells, as indicated by no increases detected in Ki67^+^, IFN-γ^+^, or CD69^+^ activating T cells in CD45^+^CD3^+^ T cells (Fig. 2g). In contrast, treatment with ICIs alone did not significantly prolong animal survival (Fig. 2b) or inhibit tumor growth (Fig. 2c), consistent with similar results observed in human clinical studies^22,23^. ICI monotherapy did not stimulate CD3^+^ infiltration (Fig. 2d) or suppress the population of myeloid cells in the tumors (Fig. 2e); it did not enhance the radio of CD8^+^/CD3^+^ T cells or affect Ki67, interferon-γ (IFN-γ), and CD69 expression in CD3^+^ T cells (Fig. 2f, g), suggesting that ICIs fail to induce activation of T cells in GBM. Notably, combination therapy with ICIs and anti-IL-6 antibody did not synergistically extended survival or reduced tumor growth, compared with single agent treatment (Fig. 2b, c). In accordance with these findings, combination therapy failed to synergistically promote cell infiltration or activation in tumor-associated T cells (Fig. 2d–g). Taken together, our data show that IL-6 neutralization enhances T-cell infiltration into GBM tumors and improves animal survival; however, it does not sensitize tumor to immune checkpoint blockade, likely due to insufficient reversal of immune suppression and limited T-cell infiltration/activation in the tumor microenvironment.

### IL-6 induces Mϕ-mediated immunosuppression but stimulates CD40 expression

**Fig. 3:**
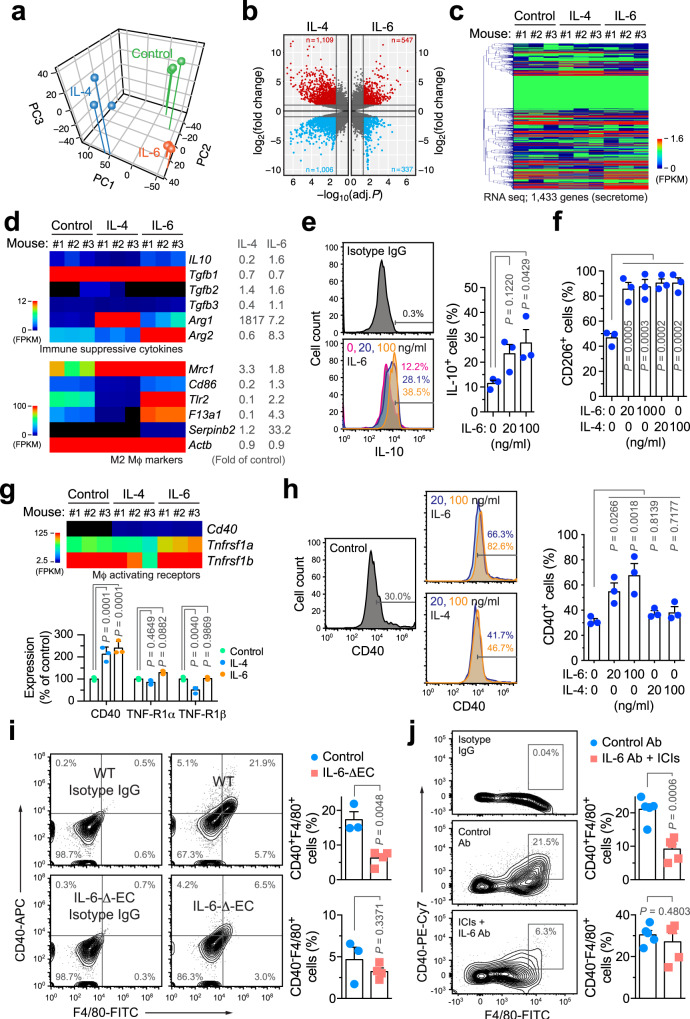
IL-6 induces Mϕ-mediated immunosuppression but stimulates CD40 expression. **a**–**e** Bone marrow (BM)-derived Mϕs were isolated from mice and treated with 50 ng/ml IL-4 and IL-6 for 2 days, followed by RNA-seq analysis (*n* = 3 mice). Genes were mapped and subjected to **a** principal component and **b** volcano plot analyses. **c** Heatmap of secretome genes. **d** Expression of immunosuppressive cytokines (top) and M2 Mϕ activation-associated genes. Left, heatmap. Right, means of fold expression of control. **e**, **f** BM-derived mouse Mϕs were treated with IL-4 and IL-6 for 2 days, and analyzed by flow cytometry. **e** IL-10 expression. Left, representative sortings. Right, quantitative results (*n* = 3 mice, mean ± SEM). Statistical analysis by one-way ANOVA with Dunnett’s test. **f** CD206 expression (*n* = 3 mice, mean ± SEM). Statistical analysis by one-way ANOVA with Dunnett’s test. **g** Expression of Mϕ activation-associated receptor genes. Left, heatmap. Right, quantitative results (*n* = 3 mice, mean ± SEM). Statistical analysis by two-way ANOVA with Dunnett’s test. **h** BM-derived mouse Mϕs were treated with IL-4 and IL-6, and analyzed by flow cytometry. Left, representative sortings. Right, quantitative results (*n* = 3 mice, mean ± SEM). Statistical analysis by one-way ANOVA with Dunnett’s test. **i** GBM was induced in control WT or IL-6-ΔEC mice. Two weeks after tumor implantation, tumor-derived single-cell suspensions were analyzed by flow cytometry (mean ± SEM, *n* = 3 mice for control group and *n* = 4 mice for IL-6-ΔEC group). Statistical analysis by two-tailed Student’s *t*-test. **j** GBM was induced in mice. Two days after treatment with IL-6 Ab and ICIs or with control Ab, tumors were analyzed by flow cytometry (*n* = 5 mice, mean ± SEM). Statistical analysis by two-tailed Student’s *t*-test. Source data are provided as a Source data file.

To explore the mechanisms by which IL-6 regulates Mϕ-mediated immunosuppression, we investigated gene expression alternations at a transcriptome level in mouse bone marrow (BM)-derived Mϕs treated with IL-6. IL-4 was used as a control, which has a well-established role for inducing alternative M2 polarization and Mϕ immunosuppression. Principal components analysis manifested a different shift toward differentiation or a lineage change induced by IL-4 or IL-6 (Fig. 3a). Moreover, IL-4- and IL-6-treated Mϕs exhibited distinct expression profiles, compared with control untreated Mϕs, as shown by volcano and heatmap plot analyses (Fig. 3b, c). Of note, specific analysis of immune-suppressive cytokines showed that IL-6 predominantly induced IL-10, TGF-β2, and arginase-1/2 expressions, whereas IL-4 robustly increased arginase-1 expression but did not enhance IL-10 expression (Fig. 3d). IL-6-induced IL-10 expression was verified by flow cytometry (Fig. 3e). Interestingly, IL-4 and IL-6 induced expression of M2 Mϕ-associated markers in a distinct manner: IL-4 seemed to selectively and markedly induce CD206 (*Mrc1*) expression, whereas IL-6 more broadly stimulated the expression of multiple markers including CD206, CD86, Toll-like receptor 2 (*Tlr2*), coagulation factor XIII (*F13a1*), and serpin family b2 (*Serpinb2*) (Fig. 3d). Flow cytometry analysis validated that both IL-4 and IL-6 enhanced CD206 expression in Mϕs (Fig. 3f). These findings collectively suggest positive roles for IL-4 and IL-6 in the induction of Mϕ M2 polarization and immunosuppression, and implicate a distinct mechanism favoring Mϕs towards immunosuppressive phenotypes by IL-6.

Multiple mechanisms mediate Mϕ activation and their co-stimulation of T cells to promote anti-tumor immunity, mainly through tumor necrosis factor (TNF) superfamilies of receptors (*Tnfrsf1a/b*) and CD40^24,25^. In contrast to our findings showing that IL-6 induces Mϕ M2 polarization and immunosuppression, our RNA sequencing (RNA-seq) data unexpectedly revealed that IL-6 stimulated expression of CD40 but not TNF-R1α/β (Fig. 3g). Moreover, IL-6, but not IL-4, consistently induced CD40 expression at both mRNA and protein levels (Fig. 3g, h). In accordance with these findings observed in BM-derived Mϕs, flow cytometry analysis showed that IL-6 substantially stimulated CD40 expression in tumor-derived Mϕs (Supplementary Fig. 3). These findings implicate a dual role of IL-6 in pro- and anti-tumor immunity, mediated through the expression of immunosuppressive cytokines and Mϕ-activating signal CD40, respectively. Furthermore, flow cytometry analysis of tumor-derived single cells showed that genetic IL-6 ablation abrogated CD40 expression in tumor Mϕs, as indicated by a decrease in CD40^+^F4/80^+^ cell population but not in CD40^−^F4/80^+^ cell population (Fig. 3i), suggesting that IL-6 is critical for positively controlling Mϕ expression of CD40 in the GBM microenvironment. Similarly, substantially reduced Mϕ expression of CD40 was observed in the tumors treated with ant-IL-6 antibody and ICIs (Fig. 3j), providing a potential mechanism for lack of therapeutic efficiency in this group, which is likely due to insufficient Mϕ activation resulted from downregulated CD40 expression.

### IL-6 induces CD40 expression through Stat3 and HIF-1α

**Fig. 4:**
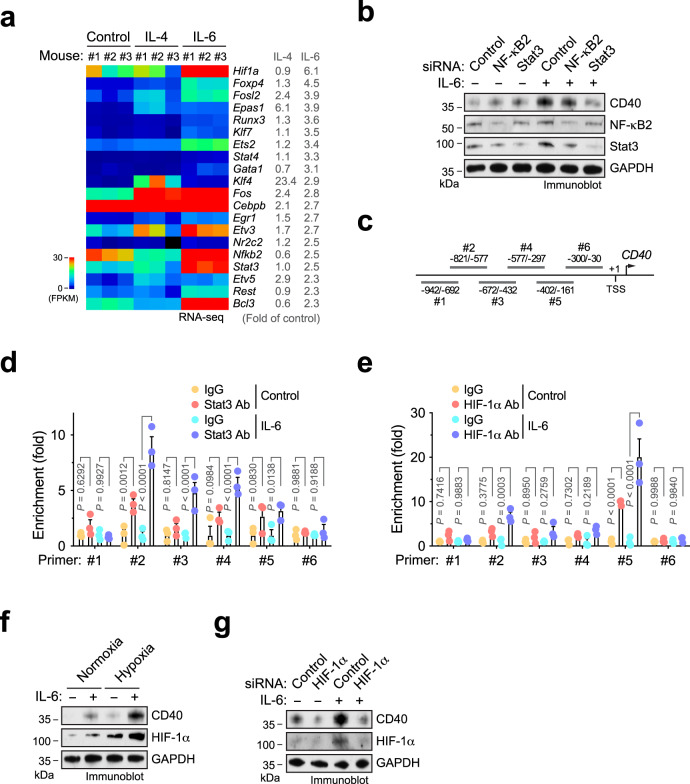
IL-6 induces CD40 expression through Stat3 and HIF-1α. **a** BM-derived Mϕs were isolated from mice and treated with 50 ng/ml IL-4 and IL-6 for 2 days, followed by RNA-seq analysis (*n* = 3 mice). Shown are top upregulated transcriptional factors induced by IL-6. Left, heatmap. Right, means of fold expression of control. **b** Human monocytes were transfected with siRNA targeting NF-κB2, Stat3, or control sequence and treated with IL-6 or control medium. Cell lysates were immunoblotted. This experiment was repeated independently twice with similar results. **c**–**e** Human monocytes were treated with IL-6 or control medium under **d** normoxia or **e** hypoxia. Nuclei protein was immunoprecipitated with **d** anti-Stat3 or **e** anti-HIF-1α antibody, or IgG, and subjected to ChIP analysis with different primers. **c** Results shown are from quantitative real-time polymerase chain reaction (RT-PCR) analysis (*n* = 3 human samples, means ± SEM). Statistical analysis by two-way ANOVA with Tukey’s test. **f** Human monocytes were treated with IL-6 or control medium under normoxia or hypoxia, followed by immunoblot analysis. This experiment was repeated independently twice with similar results. **g** Human monocytes were pretreated with siRNA targeting HIF-1α or control sequence, and treated with IL-6 or control medium under hypoxia. Cell lysates were immunoblotted. This experiment was repeated independently twice with similar results. Source data are provided as a Source data file.

Considering that the mechanisms for IL-6-induced alternative Mϕ polarization have been well defined^21,26,27^, we focused our study on the molecular mechanism by which IL-6 regulates CD40 expression. Computational bioinformatics analysis of top 20 upregulated transcription factors revealed that IL-6 exclusively induced hypoxia-inducible factor (HIF)-1α, Ets2, NK-κB2, Stat3, and Bcl3 expression to a robust level (fragments per kilobase million (FPKM) > 1.0, by RNA-seq), compared to control untreated and IL-4-treated mouse Mϕs (Fig. 4a). We initially focused our study on HIF-1α, NK-κB, and Stat3 that are known to participate IL-6 signaling regulation^28–31^. Small interfering RNA (siRNA)-mediated knockdown of Stat3, but not of NK-κB2, robustly abrogated IL-6-induced CD40 expression in human Mϕs (Fig. 4b). Furthermore, chromatin immunoprecipitation (ChIP) analysis showed that Stat2 interacts with CD40 promoter, particularly in the region from −821 to −577 downstream of transcription start site (TSS), in an IL-6-inducible manner (Fig. 4c, d), collectively suggesting that Stat3 is critical for IL-6-induced CD40 transcription.

Considering that HIF-1α, a master regulator of cell responses to hypoxia, was identified at the top of the IL-6-inducible transcriptional factors (Fig. 4a), we investigated the role of HIF-1α in IL-6-induced CD40 expression under hypoxia. Our data showed that HIF-1α binds to CD40 promoter in the region from −402 to −161 downstream of TSS under hypoxia, and IL-6 robustly enhanced this binding (Fig. 4c, e). Furthermore, immunoblot analysis indicated that hypoxia stimulates IL-6-induced CD40 expression (Fig. 4f). siRNA-mediated HIF-1α knockdown abolished the IL-6-induced CD40 expression (Fig. 4f, g). Together, our data reveal that IL-6 induces CD40 expression through Stat3 and HIF-1α.

### IL-6 neutralization and CD40 stimulation sensitizes GBM to immune checkpoint blockade

**Fig. 5:**
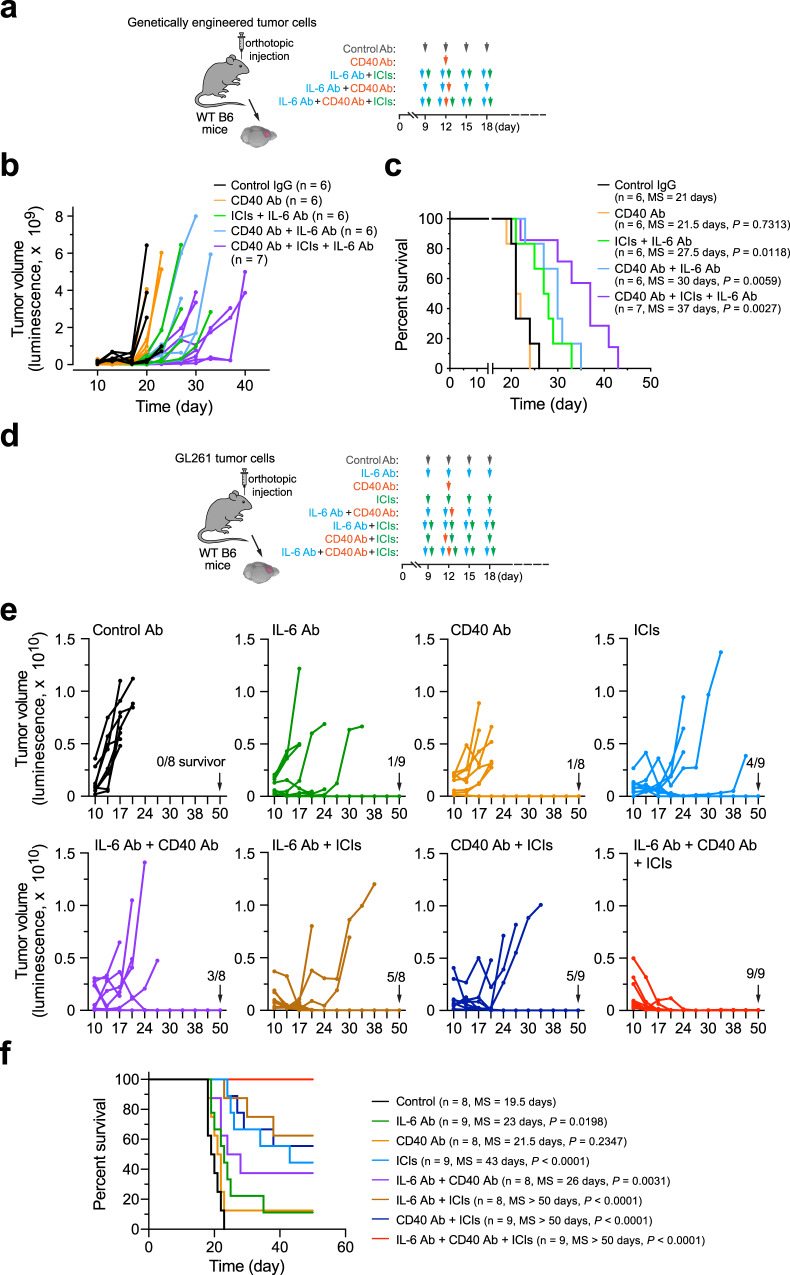
IL-6 neutralization and CD40 stimulation sensitizes GBM to immune checkpoint blockade treatment. GBM was induced in mice by transplantation with **a**–**c**, tumor cells derived from RCAS-genetically engineered model (*n* = 6–7 mice, specific *n* numbers are shown in the figure) or **d**–**f** GL261 tumor cells (*n* = 8–9 mice, specific *n* numbers are shown in the figure), followed by different treatment and survival analyses. **a**, **d** Experimental procedure. **b**, **e** Tumor volume was analyzed by bioluminescence imaging. **c**, **f** Mouse survival was monitored and analyzed by two-sided Log-rank Mantel–Cox analysis. MS, median survival. Source data are provided as a Source data file.

Given a dual role of IL-6 in immunosuppressive cytokine-mediated pro-tumor effects immunity and CD40-mediated anti-tumor immunity (Fig. 3), we next sought to test experimental therapy that combines CD40 agonist to maximize tumor immunity in anti-IL-6 treatment. GBM was genetically induced in wild-type (WT) mice, followed by treatment with IL-6-neutralizing antibody, CD40 agonist antibody, and ICI, alone or combined (Fig. 5a). Our data showed that CD40 stimulation alone did not affect tumor growth (Fig. 5b) or animal survival (Fig. 5c). In a parallel study, combination therapy with a CD40 agonist plus checkpoint inhibitors also did not extend animal survival (Supplementary Fig. 4). However, dual therapy that combines IL-6 neutralization and CD40 stimulation markedly sensitized GBM to ICI treatment, as triple treatment (CD40 antibody, IL-6 antibody, and ICIs) substantially delayed tumor growth (Fig. 5b) and enhanced mouse survival with an almost doubled median survival (37 days), compared with 21 days in control IgG-treated mice (Fig. 5c). These results suggest dual-targeting IL-6 and CD40 as an efficient strategy for overcoming GBM resistance to ICI treatment.

In addition, we tested therapeutic efficacy of the dual-targeting treatment in an independent GL261 mouse GBM model (Fig. 5d). Similar to the findings observed in the genetically induced GBM model, pro-CD40 and anti-IL-6 monotherapy showed limited and moderate therapeutic effects on tumor growth and animal survival, respectively (Fig. 5e, f). Consistent with previously published data^32^, GL261 tumors partially responded to ICI treatment. Notably, combination of ICIs with the dual-targeting IL-6 and CD40 resulted in complete therapeutic responses, as indicated by all treated animal survived at the end time point without detectable tumors (Fig. 5e, f).

### IL-6 neutralization and CD40 stimulation plus immune checkpoint blockade synergistically reduces Mϕ-mediated immune suppression and enhances T-cell infiltration and activation in GBM

**Fig. 6:**
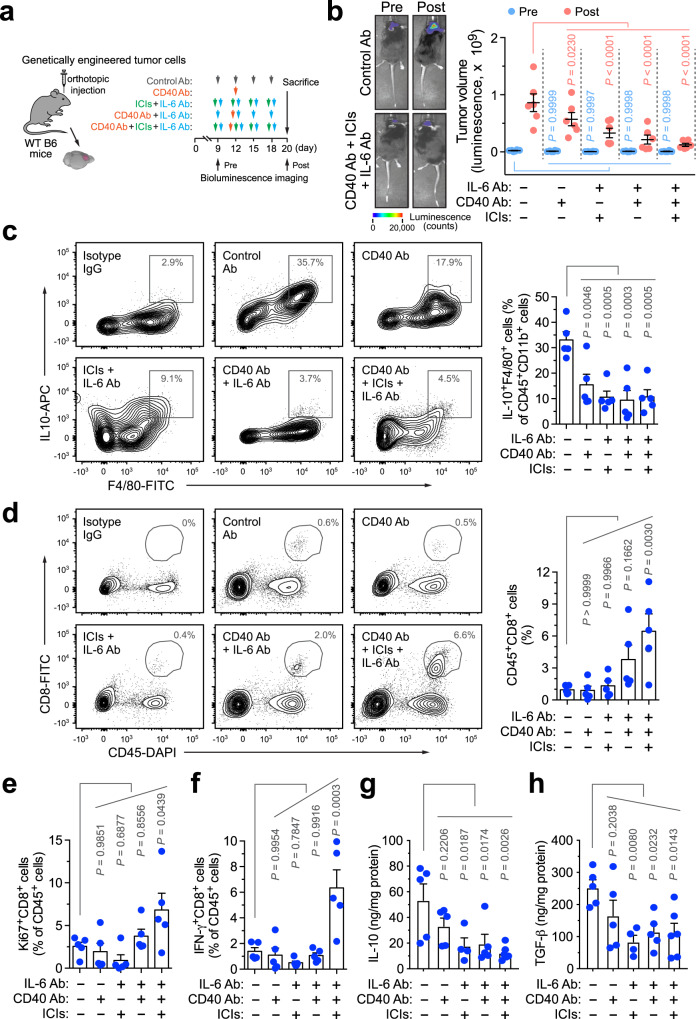
IL-6 neutralization and CD40 stimulation plus immune checkpoint blockade synergistically reverses Mϕ-mediated immune suppression and activates GBM-associated T cells. GBM was induced in mice, followed by different treatment and endpoint analyses. **a** Experimental procedure. **b** Tumor volume was analyzed pre- and post treatment by bioluminescence imaging. Left, representative images. Right, quantified results (*n* = 6 mice, mean ± SEM). Statistical analysis by two-way ANOVA with Dunnett’s test. **c**–**f** Tumor-derived single-cell suspensions were analyzed by flow cytometry. **c**, **d** Cells were probed with **c**, anti-F4/80 and anti-IL-10, or **d** anti-CD45 and anti-CD8 antibodies. Left, representative sortings. Right, quantified results (*n* = 5 mice, mean ± SEM). Statistical analysis by one-way ANOVA with Dunnett’s test. **e**, **f** Cells were probed with **e** anti-CD8 and anti-Ki67, or **f** anti-CD8 and anti-IFN-γ antibodies. Quantified results are shown (*n* = 5 mice, mean ± SEM). Statistical analysis by one-way ANOVA with Dunnett’s test. **g**, **h** Tumor lysates were subjected to **g** IL-10 and **h** TGF-β ELISA analysis (mean ± SEM, *n* = 4 mice for ICI plus IL-6 Ab treatment group, *n* = 6 mice for ICIs, CD40 Ab, plus IL-6 Ab treatment group, and *n* = 5 mice for other groups). Statistical analysis by one-way ANOVA with Dunnett’s test. Source data are provided as a Source data file.

We next investigated the effects of the combination therapy on tumor immunity 2 days after treatment in the genetically engineered GBM model (Fig. 6a). Our data indicated that the triple treatment almost completely blocked tumor growth during the therapy window and, to a lesser extent, combination therapy with IL-6 neutralization and CD40 stimulation markedly reduced tumor growth (Fig. 6b). Flow cytometry analysis of tumor-derived single-cell suspensions showed that all treated groups, compared with control IgG-treated group, exhibited reversed immunosuppressive activity of tumor-associated Mϕs, evidenced by the reduced populations of IL10^+^F4/80^+^ cells by up to 70% (Fig. 6c). However, only triple treatment induced a significant (*P* < 0.01), robust sevenfold increase in the infiltrates of cytotoxic CD45^+^CD8^+^ T cells (Fig. 6d), which likely induced the therapeutic benefits including delayed tumor growth and extended animal survival (Fig. 5b, c). Furthermore, triple treatment enhanced activities of these infiltrated T cells, as indicated by the increases in CD45^+^CD8^+^Ki67^+^ and CD45^+^CD8^+^IFN-γ^+^ cell populations that express proliferative marker Ki67 and cytotoxic cytokine IFN-γ (Fig. 6e, f). In addition, all treatments, except for CD40 stimulation treatment alone, reduced the expression of immunosuppressive cytokines including IL-10 and TGF-β, in the tumor tissues, compared with control treatment with IgG (Fig. 6g, h), verifying the reversal of Mϕ–mediated immune suppression in GBM.

In a parallel study with the GL261 GBM model (Supplementary Fig. 6a), triple treatment similarly decreased the populations of IL-10^+^F4/80^+^ Mϕs (Supplementary Fig. 6b), enhanced the infiltration of CD45^+^CD8^+^ T cells into the tumors (Supplementary Fig. 6c), and increased the populations of CD45^+^CD8^+^IFN-γ^+^ active T cells (Supplementary Fig. 6d). Taken together, our data suggests that combination therapy with dual-targeting IL-6 and CD40 with checkpoint blockade robustly reverses Mϕ-mediated immune suppression, leading to T-cell infiltration and activation in GBM.

### High IL-6 expression and low CD40 expression correlate with poor survival in human patients with GBM

**Fig. 7:**
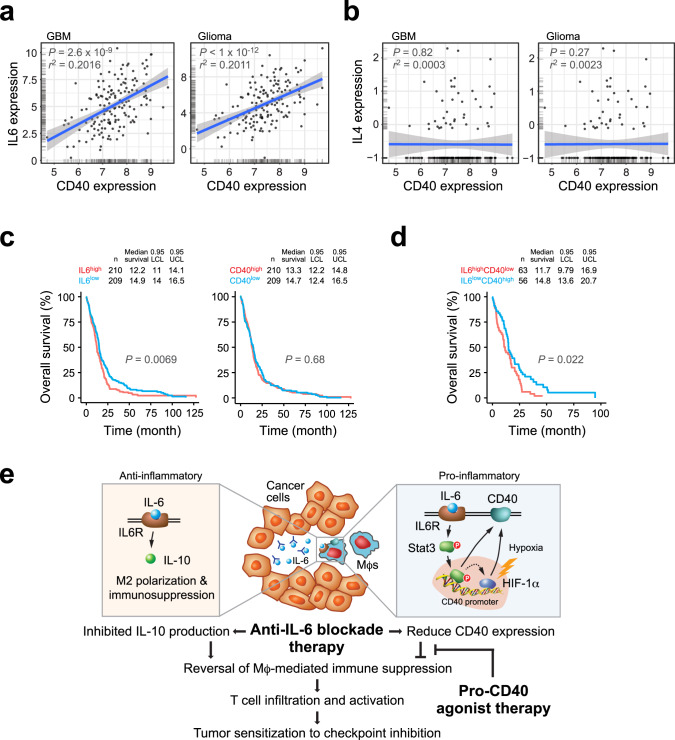
High IL-6 expression and low CD40 expression correlate with poor survival in human GBM patients. **a**, **b** Correlation of CD40 expression with **a** IL-6 and **b** IL-4 expression was subjected to linear regression analyses using GlioVis/TCGA GBM-RNA-seq (*n* = 160 patients) and low-grade glioma (*n* = 513 patients) data sets. Statistical analysis by linear regression analysis. **c**, **d** Correlation of IL-6 and CD40 expression (high/low cutoff of 40%) with overall survival was analyzed using TCGA-Firehose data set. Statistical analysis by two-sided log-rank test. **e** A schematic model. IL-6 induces anti-inflammatory and pro-inflammatory functions in Mϕs, through IL-10 and CD40 expression, respectively. Combination therapy by anti-IL-6 neutralization and CD40 activation reverses Mϕ-mediated tumor immunosuppression and promotes T-cell infiltration and activation, sensitizing tumor to checkpoint inhibition treatment.

We finally analyzed The Cancer Genome Atlas (TCGA) data sets to investigate the role of IL-6 and CD40 in human patients with GBM or glioma. In accordance with our results showing (1) IL-6 stimulated CD40 expression in Mϕs in vitro (Fig. 3g, h) and (2) IL-6 knockout or inhibition reduced CD40 expression in tumor-associated Mϕs in mice (Fig. 3i, j), linear regression analysis of gene expression in TCGA data revealed that IL-6 expression correlated with CD40 expression in patients with both GBM and glioma (Fig. 7a). Consistent with the pro-inflammatory role of IL-6 in GBM, CD40 expression also correlated with expression of pro-inflammatory cytokines including TNF-α, IL-1α, and IL-1β (Supplementary Fig. 7a). In contrast, expression of IL-4, another important cytokine that regulates Mϕ and T-cell functions similar to IL-6, did not correlate with CD40 expression in patients with GBM or all grades of glioma (Fig. 7b). Furthermore, clinical overall survival data suggested that high IL-6 expression was associated with poor survival in patients with GBM (Fig. 7c and Supplementary Fig. 7b), whereas CD40 expression was not a prognostic factor in survival rates of GBM patients (Fig. 7c and Supplementary Fig. 7b). Interestingly, those GBM patients with higher IL-6 expression and lower CD40 expression exhibited worst overall survival (Fig. 7d and Supplementary Fig. 7b). These clinical data together support our experimental results showing that IL-6 controls CD40 expression in GBM-associated Mϕs, and that IL-6 and CD40 critically regulates tumor immunity and determine pathological outcomes.

In sum, our work uncovers a complex role for IL-6 in regulation of tumor immunity (Fig. 7e): IL-6 induces alternative Mϕ polarization and anti-inflammatory immune suppression, whereas it stimulates pro-inflammatory CD40 expression via Stat3/HIF-1α in GBM. As such, anti-IL-6 monotherapy fails to induce a robust anti-tumor activity and to overcome GBM resistance to checkpoint blockade, likely due to reduced co-stimulatory CD40 signal and insufficient T-cell infiltration and activation. Based on these results, we develop dual-targeting anti-IL-6 and pro-CD40 strategy to activate tumor immunity, sensitizing tumor to T-cell-based immunotherapies including checkpoint blockade.

## Discussion

Activation of tumor-associated T cells by immune checkpoint blockade has been one of the most successful immunotherapy approaches for various solid tumors^33,34^; however, checkpoint inhibitors have been largely ineffective in treating immunologically cold tumors including GBM^4,35^. As such, the PD-1 inhibitor nivolumab and CTLA-4 inhibitor ipilimumab have shown little or no overall survival benefits in recent GBM clinical trials, mainly due to the lack or paucity of T-cell infiltrates in the immunosuppressive microenvironment^22,23^. Here we develop a dual-targeting anti-IL-6 and pro-CD40 strategy to reverse Mϕ-mediated tumor immunosuppression and to overcome primary tumor resistance to immune checkpoint blockade in GBM.

Tumor-associated Mϕs, accounting for more than half of the immune cell population in GBM, induce a wide variety of immunosuppressive functions^20,36,37^. Growing evidence suggests that tumor Mϕs are an attractive target in GBM immunotherapy, in particular for improving checkpoint blockade treatment^38–43^. IL-6, albeit initially recognized as a pro-inflammatory cytokine^28,44^, has recently been shown to stimulate alternative Mϕ activation, a well-known process associated with anti-inflammatory immunosuppressive functions, in diabetic and obesity conditions^26,27^. Consistent with previous work showing that tumor stroma secretes IL-6 to inhibit anti-tumor immunity^45^, our recent study revealed that tumor vascular niche-derived IL-6 induces alternative Mϕ activation^21^. Here, our study demonstrates that IL-6 blockade partially reverses tumor immunosuppression and stimulates T-cell infiltration into GBM tumors. Importantly, our work by unbiased transcriptome analysis validates that IL-6 drives alternative Mϕ activation and expression of immunosuppressive IL-10, mediated through a genetic reprogramming process that is distinct from the mechanism induced by IL-4, a well-known inducer of alternative Mϕ activation^17,46^. Furthermore, recent work shows that IL-6 contributes to systemic dysfunction of dendritic cells in pancreatic cancer, suggesting additional benefits of anti-IL-6 therapy^47^. However, our work shows that anti-IL-6 therapy alone has only modest efficacy and does not synergize with checkpoint inhibitors, suggesting that anti-IL-6 monotherapy may not fully reverse Mϕ-mediated immunosuppression to activate anti-tumor immunity. Similarly, a recent study shows that anti-IL-6 therapy induces a similar therapeutic benefit but the anti-tumor activity is additive with PD-1 inhibition, due to a checkpoint inhibition-sensitive murine GBM model used in the study^32^. In fact, anti-IL-6 monotherapy in clinics does not exert robust therapeutic benefits in multiple types of cancers^48–50^. Our further work identifies a role of CD40 for tumor resistance to anti-IL-6 and checkpoint blockade treatments.

CD40, a member of the TNF receptor family, is a co-stimulatory protein that plays a crucial role in pro-inflammatory immune activation of antigen-presenting cells such as dendritic cells and Mϕs in cancer^24,51–53^. Previous studies show that CD40 stimulation activates tumor-associated Mϕs or T cells and inhibits tumor progression in melanoma, lymphoma, and pancreatic carcinoma^54–56^. Likewise, CD40 agonist therapy reprograms the tumor microenvironment and sensitizes the tumor to checkpoint blockade treatment in breast and pancreatic cancers, and osteosarcoma^57–59^. However, consistent with recent reports showing that antibody-based CD40 activation monotherapy only slightly affects animal survival in syngeneic mouse GBM models, particularly in GL261 models^60,61^, our study indicates that CD40 antibody treatment alone has no therapeutic efficacy and also fails to sensitize checkpoint blockade therapy in our genetically engineered GBM model, implicating that multiple mechanisms for Mϕ activation exist in GBM. Notably, our RNA-seq data identified a complex role of IL-6 in regulating Mϕ activity, including both pro-inflammatory (stimulation of CD40 expression) and anti-inflammatory (induction of alternative Mϕ activation and immunosuppression) functions, which led us to explore dual-targeting anti-IL-6 and pro-CD40 therapy to maximally activate Mϕ-mediated anti-tumor immunity. Our data show that this dual-targeting strategy substantially reverses Mϕ-mediated tumor immunosuppression and induces infiltration of CD8^+^ T cells into the tumors. Consistent with these findings, a recent study shows that blockade of IL-6 receptor and activation of CD40 by adenovirus-based gene therapy markedly prolongs animal survival and inhibits the expression of immunosuppressive cytokine TGF-β in pancreatic cancer^62^. Strikingly, our work further shows that this dual-targeting anti-IL-6 and pro-CD40 strategy overcomes GBM resistance to checkpoint blockade therapy, likely due to a shift of tumor immune status from immunologically cold to hot. We expect that optimization of the dose and timing of this dual-targeting therapy will further enhance therapeutic efficacy in GBM.

IL-6 is a pleiotropic cytokine that regulates immune and inflammatory responses mainly through inducing activation of Jak/STAT-3 and Ras/Erk/C/EBP pathways in immune cells^28,29^. Our work uncovers that IL-6 induces CD40 expression via Stat3 in Mϕs. Previous work shows that engagement of CD40 induces Jak/Stat3 phosphorylation and activation^63^, implicating an IL-6-inducible positive feedback loop that activates Stat3/CD40 signals in Mϕs. Furthermore, we also identify that HIF-1α, a master regulator of hypoxia-induced cell responses, is critical for IL-6-induced CD40 expression in Mϕs under hypoxia, as both Stat3 and HIF-1α bind to CD40 promoter upon IL-6 stimulation. Interestingly, it is well known that hypoxia induces Stat3 activation, and that Stat3 stabilizes HIF-1α via protein–protein interaction^64–66^, suggesting that IL-6 activates Stat3 to activate HIF-1α and further enhance CD40 expression in Mϕs under hypoxia. These results illustrate a hypoxia-inducible mechanism by which IL-6 induces pro-inflammatory CD40 expression through Stat3 and HIF-1α, in addition to the role for IL-6 in anti-inflammatory alternative Mϕ activation and tumor immunosuppression. Our work shows that hypoxia enhances IL-6-mediated CD40 expression in tumor Mϕs, implicating that cancer therapy by modulation of tumor metabolism or normalization of blood vessels to relieve tumor hypoxia may need additional CD40 agonist treatment to stimulate anti-tumor immunity.

In summary, our work unravels an IL-6-regulated cellular mechanism that controls Mϕ-mediated tumor immunity through IL-10 and Stat3/HIF-1α/CD40 expression. Our findings suggest that dual-targeting IL-6 and CD40 may offer exciting opportunities for reversing Mϕ-mediated tumor immunosuppression and improving T-cell-based immunotherapy against GBM. This dual-targeting treatment may serve as an adjuvant therapy after standard of care, including surgery and radiochemotherapy, which reduce tumor burden and induce immunogenic cell death in GBM. Of note, our work shows that dual-targeting IL-6 and CD40 plus ICIs completely eradicate GL261 tumors in all treated mice, suggesting that cocktail immunotherapy combining ICIs with neutralizing antibodies against IL-6 or IL-6 receptor, such as tocilizumab or sarilumab, and anti-CD40 agonist antibodies, such as APX005M, may act as an effective therapeutic approach for GBM, and possibly for other immunologically cold tumors, such as pancreatic, ovarian, and prostate cancers, which are characterized by a prominent infiltration of immunosuppressive Mϕs.

## Methods

### Human monocyte isolation and treatment

Primary human monocytes were provided by Human Immunology Core at the University of Pennsylvania. Peripheral blood mononuclear cells were collected from healthy human volunteer donors and monocytes were isolated following leukapheresis by negative selection. All specimens were collected under a University of Pennsylvania Institutional Review Board-approved protocol and written informed consent was obtained from each donor. We have complied with all relevant ethical regulations for work with human participants. Cells were incubated in RPMI-1640 medium supplemented with 5% fetal bovine serum (FBS) and treated with 10 ng/ml human CSF-1 (BioLegend, 574806) and 100 ng/ml human IL-6 (BioLegend, 570808).

### Mice

WT mice on the C57BL/6J background were obtained from Jackson Lab. *Cdh5-Cre*^ERT2^;*Il6*^fl/fl^ mice were generated by crossing *Il6*^fl/fl^ mice with *Cdh5-Cre*^ERT2^ mice^67^. Mice (2 weeks old) were intraperitoneally injected with 0.1 ml of 5 mg/ml tamoxifen daily for consecutive 5 days. All animals were housed at room temperature with a 12 h-light/12 h-dark cycle in the Association for the Assessment and Accreditation of Laboratory Animal Care-accredited animal facility of the University of Pennsylvania. All animal studies were reviewed and approved by the Institutional Animal Care and Use Committees at the University of Pennsylvania. We have complied with all relevant ethical regulations for animal testing and research.

### Mouse BMDM isolation and treatment

Mouse BM-derived macrophages (BMDMs) were isolated^21^. Freshly isolated femur and tibia from WT C57BL/6 mice were flushed with RPMI-1640 medium (Life Technologies). Cells were collected and passed through a 40 μm strainer. Red cells were depleted with ACK lysis buffer (Thermo Fisher). BM cells were cultured in RPMI-1640 medium supplemented with 5% FBS (Life Technologies). Cells were incubated with 10 ng/ml mouse CSF-1 (Biolegend, 576404) for 3 days, to induce macrophage differentiation, followed by treatment with 10 ng/ml mouse CSF-1 in the presence or absence of 100 ng/ml mouse IL-4 (Biolegend, 574306) or IL-6 (Biolegend, 575708) for 4 days.

### RNA-seq analysis

Treated BMDM were lysed in TRIzol (Thermo Fisher) and RNA was extracted according to the manufacturer’s instructions, followed by RNA purification using an RNeasy Plus Mini Kit (Qiagen). DNA library was constructed with a TruSeq mRNA Stranded Kit (Illumina). The RNA from each step and library DNA quality were analyzed with RNA Nano assay chips, RNA Pico assay chips, and DNA Nano assay chips using a 2100 bioanalyzer (Agilent). Library was subjected to next-generation sequencing analysis in a high-throughput sequencing center with a HiSeq2500 at the Children’s Hospital of Philadelphia/Beijing Genomics Institute core facility. The sequences were aligned to the GRCm38 reference genome using RNA-Star (v2.4.2a; https://github.com/alexdobin/STAR). The gene expression was normalized and calculated as FPKM values by Cufflinks (v2.2.1) (http://cole-trapnell-lab.github.io/cufflinks/releases/v2.2.1/) with Gencode M5 gene annotations (https://www.gencodegenes.org/mouse/release_M5.html).

### GBM tumor induction and treatment

A genetically engineered mouse GBM model was induced^68^. Briefly, chicken DF-1 fibroblasts (American Type Culture Collection) were transfected with RCAS-PDGF-B and RCAS-Cre plasmids, followed by orthotopically injecting into *Ntv-a*;*Ink4a-Arf*^−/−^;*Pten*^fl/fl^;*LSL-Luc* mice. Tumors were freshly isolated and subjected to mechanical dissociation with a gentleMACS Dissociator (Miltenyi). Enzymatic digestion with collagenase II and dispase II were performed to obtain single-cell suspensions, followed by culture in mouse stem cell medium (Stemcell Technologies) for collecting tumor spheres. Eight-week old *Cdh5-Cre*^ERT2^;*Il6*^fl/fl^ or WT C67BL/6 mice (half male and half female) were stereotactically injected with 3 × 10^5^ GBM tumor cells. For the syngeneic GBM model, 2 × 10^5^ GL261 glioma cells were orthotopically injected into 8-week-old WT C57/B6 mice (half male and half female). Tumor-bearing mice were intraperitoneally treated with anti-PD-1 (200 μg/mouse, BioXcell, BE0146), anti-CTLA-4 (200 μg/mouse, BioXcell, BE0131), CD40 (100 μg/mouse, BioXcell, BE0016-2), or anti-IL-6 (200 μg/mouse, BioXcell, BE0046) antibody or control rat IgG (BioXcell, BE0090), respectively. Tumor volume was monitored by whole-body bioluminescence using an IVIS 200 Spectrum Imaging System after retro-orbital injection of luciferin (150 mg/kg, GoldBio). Post-injection survival was monitored for 50 days. Mice were killed when exhibiting severe GBM symptoms including domehead, hemiparesis, or >20% of body weight loss.

### Mass cytometer (CyTOF)

Single-cell suspensions derived from freshly isolated tumors were prepared by mechanical dissociation with a gentleMACS Dissociator (Miltenyi Biotech) and enzymatic digestion with collagenase II and dispase II. Cells were incubated with 25 μM cisplatin, followed by staining at room temperature for 30 min with heavy metal-conjugated antibodies provided by CyTOF core at the Penn Institute for Immunology. Cells were fixed with 1.6% paraformaldehyde and stained with Cell-ID Intercalator-Ir (Fluidigm) and analyzed using a CyTOF mass cytometer (Fluidigm), followed by analysis with Cytobank software (7.3.0).

### Flow cytometry

Single-cell suspensions derived from tumors were stained with fluorescence dye-conjugated antibodies against CD3 (1 : 100, BioLegend, 100233), CD11b (1 : 200, BioLegend, 101228), CD11b (1 : 200, eBioscience, 69-0112-80), CD11c (1 : 200, eBioscience, 17-0114-81), MHCII (1 : 100, eBioscience, 47-5321-80), CD45 (1 : 200, eBioscience, 48-0451-82), B220 (1 : 100, eBioscience, 47-0452-80), Gr1 (1 : 200, BioLegend, 108415), CD8a (1 : 100, BioLegend, 100706), IFN-g (1 : 100, BioLegend, 505808), Ki67 (1 : 100, BioLegend, 652405), IL-10 (1 : 100, BioLegend, 505009), CD40 (1 : 100, BioLegend, 124621), NK1.1 (1 : 100, BioLegend, 108707), CD4 (1 : 100, BioLegend, 100540), Ly6G (1 : 200, BioLegend, 127615), Ly6C (1 : 200, BioLegend, 128005), CD69 (1 : 200, BioLegend, 104511), F4/80 (1 : 200, BioLegend, 123107), CD206 (1 : 100, BioLegend, 141710), CD40 (1 : 100, BioLegend, 124611), or control IgG. Cells were analyzed using Accuri C6 (BD Biosciences) and FACSCanto II flow cytometers (BD Biosciences) and FlowJo software (V9).

### Enzyme-linked immunosorbent assay

Mouse tumor tissues were homogenized with extraction buffer. The supernatant was analyzed using mouse IL-10 (Biolegend, 431417) or TGF-β ELISA kits (Biolegend, 433007) according to the manufacturer’s instructions.

### Isolation of tumor-associated myeloid cells

Single-cell suspensions derived from tumors were incubated with anti-CD11b antibody- conjugated microbeads (1 : 100, Miltenyi Biotech, 130-049-601) for 15 min at 4 °C and separated by magnetic-activated cell sorting (MS) column with a separator. The eluted cells were cultured in RPMI-1640 medium with 10% FBS.

### siRNA treatment

Human monocytes were transfected with siRNAs targeting HIF-1α (Thermo Fisher, 42840), Stat3 (Life Technologies, 4390824), nuclear factor-κB (NF-κB) (Thermo Fisher, 106835), or control siRNA (Qiagen, 1027280) using Amaxa 4D-Nucleofector (Lonza) with program EA-100.

### Chromatin immunoprecipitation

ChIP assays were performed using a Magna ChIP kit (Millipore, MAGNA0001)^69,70^. In brief, treated human monocytes/macrophages (10^7^ cells cultured in 15 cm dishes) were crosslinked with 1% formaldehyde for 10 min at room temperature, followed by glycine incubation for 5 min. Nucleic lysis were sonicated for four cycles (each for 8 × 2 s, interval 45 s) using a W-385 sonicator (Heat Systems Ultrasonics). Immunoprecipitation was conducted using 20 μg anti-Stat3 (Cell Signaling, 12640) or anti-HIF-1α (Cell Signaling, 14179) antibody, or 20 μg anti-rabbit IgG (Santa Cruz, sc-2027) with protein A-conjugated beads. Inputs, acquired from 1% sheared DNA, and immunoprecipitants were reverse-crosslinked and purified. The primers pairs for CD40 promoter used in ChIP–quantitative reverse-transcriptase PCR are listed as follows: Primer #1: forward primer (FP), 5′-agtcttgctctgccttcgag-3′, reverse primer (RP), 5′-cgcctgtaatccagcacttt-3′; Primer #2: FP, 5′-aacgccactacatccggtta-3′, RP, 5′-cgtctcaacttcccatccat-3′; Primer #3: FP, 5′-ggccccactcttaataaatgc-3′, RP, 5′-acaccaccacgcagaaaac-3′; Primer #4: FP, 5′-atggatgggaagttgagacg-3′, RP, 5′-aggagctagcctgcttcctg-3′; Primer #5: FP, 5′-cggttctgccaggataccta-3′, RP, 5′-taattcccccgggagtttag-3′; and Primer #6: FP, 5′-gtcgcaggaagcaggcta-3′, RP, 5′- cgaggcctctgctgactc-3′.

### Immunoblotting

Cells were lysed with an NP-40 buffer containing protease inhibitor cocktail (Roche, 11697498001). Protein (20 μg) was resolved by 4–15% SDS-polyacrylamide gel electrophoresis (Bio-Rad). After transfer, polyvinylidene difluoride membranes were blotted with anti-HIF-1α (1 : 1000, Cell Signaling, 14179), anti-Stat3 (1 : 1000, Cell Signaling, 12640), anti-NF-κB (1 : 1000, Cell Signaling, 4882), anti-CD40 (1 : 1000, Cell Signaling, 86165), or anti-GAPDH (1 : 3000, Cell Signaling, 5174) antibody overnight at 4 °C. Proteins were detected with goat anti-rabbit or anti-mouse IgG-HRP conjugate (1 : 5000, Bio-Rad, 1706515 or 1706516), followed by ECL development (GE Healthcare, RPN2232). Full scan images of blots are available as a Source data file.

### Statistical analysis

All grouped data were presented as box plot in figures. Statistical analysis was performed using Student’s *t*-test or analysis of variance for experiments with two groups or more than two groups, respectively. Kaplan–Meier survival curves were generated using Prism software and log-rank test was performed to assess statistical significance between groups in mouse experiments. The survival analysis of TCGA (Glioblastoma, Firehose Legacy) and GlioVis data sets was conducted using R software (Version 3.6.3). A two-sided *P*-value < 0.05 was considered significant.

### Reporting summary

Further information on research design is available in the Nature Research Reporting Summary linked to this article.

## Supplementary information

Supplementary Information

Reporting Summary

## Data Availability

RNA-seq data have been deposited in NCBI’s Gene Expression Omnibus (GSE151213). Gene expression and survival data of glioma patients were obtained from TCGA (https://portal.gdc.cancer.gov) and GlioVis (http://gliovis.bioinfo.cnio.es/). The remaining data are available within the Article, Supplementary Information, or available from the authors upon request. Source data are provided with this paper.

## Source data

Source data_raw data
