# Supplementary material for: Synergistic immunotherapy of glioblastoma by dual targeting of IL-6 and CD40
Source: Nat Commun. 2021 Jun 8;12:3424. doi: 10.1038/s41467-021-23832-3 (PMC8187342; doi:10.1038/s41467-021-23832-3)
Supplement: Supplementary file 1 — Supplementary Information [file 41467_2021_23832_MOESM1_ESM.pdf]

Supplementary Information for

Synergistic immunotherapy of glioblastoma by dual targeting of IL-6 and CD40

Fan Yang[1,#], Zhenqiang He[1,#], Hao Duan[1,#], Duo Zhang[1], Juehui Li[1], Huijuan Yang[1], Jay F. Dorsey[1], Wei Zou[1], S. Ali Nabavizadeh[2], Stephen J. Bagley[3], Kalil Abdullah[4], Steven Brem[3,4], Lin Zhang[5], Xiaowei Xu[6], Katelyn T. Byrne[3], Robert H. Vonderheide[3,7,*], Yanqing Gong[8,*], & Yi Fan[1,3,4,7,*]

[1]Department of Radiation Oncology, University of Pennsylvania, Philadelphia, PA, USA
[2]Department of Radiology, University of Pennsylvania, Philadelphia, PA, USA
[3]Abramson Cancer Center, University of Pennsylvania, Philadelphia, PA, USA
[4]Department of Neurosurgery, University of Pennsylvania, Philadelphia, PA, USA
[5]Department of Obstetrics and Gynecology, University of Pennsylvania, Philadelphia, PA, USA
[6]Department of Pathology and Laboratory Medicine, University of Pennsylvania, Philadelphia, PA, USA
[7]Institute for Immunology, University of Pennsylvania, Philadelphia, PA, USA
[8]Division of Human Genetics and Translational Medicine, Department of Medicine, University of Pennsylvania, Philadelphia, PA, USA

[#]These authors contributed equally

*Correspondence to: rhv@upenn.edu; gongy@pennmedicine.upenn.edu; yi.fan@pennmedicine.upenn.edu

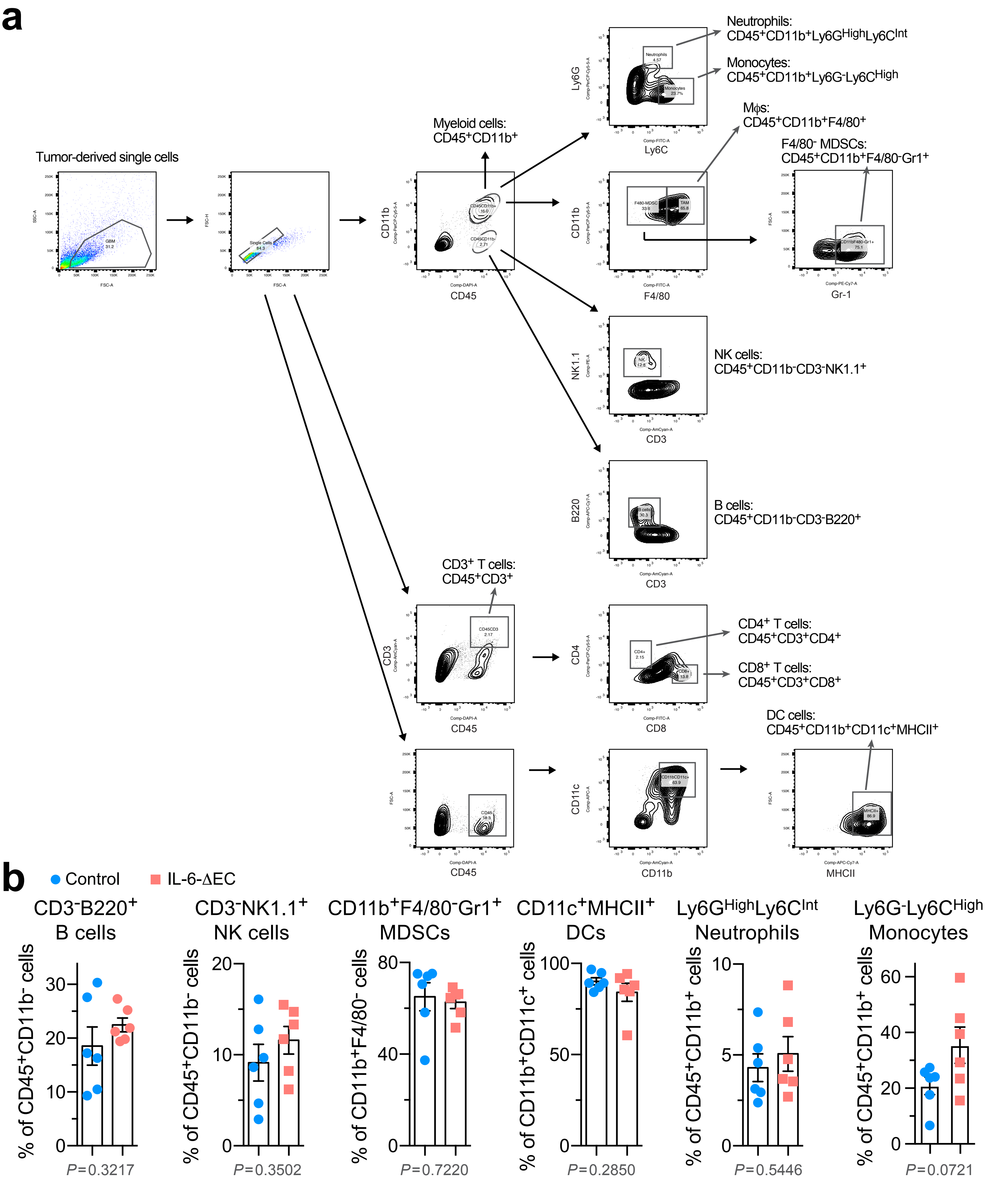

Supplementary Fig. 1. Effects of genetic ablation of IL-6 on GBM immunity.
GBM was induced by RCAS-mediated genetic engineering, followed by orthotopic tumor implantation into *Cdh5-Cre*^{ERT2};*Il6*$^{fl/fl}$ recipient mice that were pre-treated with (IL-6-ΔEC) or without (Control) tamoxifen. Two weeks after tumor implantation, tumors were excised. Tumor-derived single cell suspensions were analyzed by flow cytometry. **a**, Sorting strategies used for the assays prsented in this figure and Fig.1d-f. **b**, Analysis for different immune cells (n = 6 mice, mean ± SEM). Statistical analysis by two-tailed Student's *t* test. Source data are provided as a Source data file.

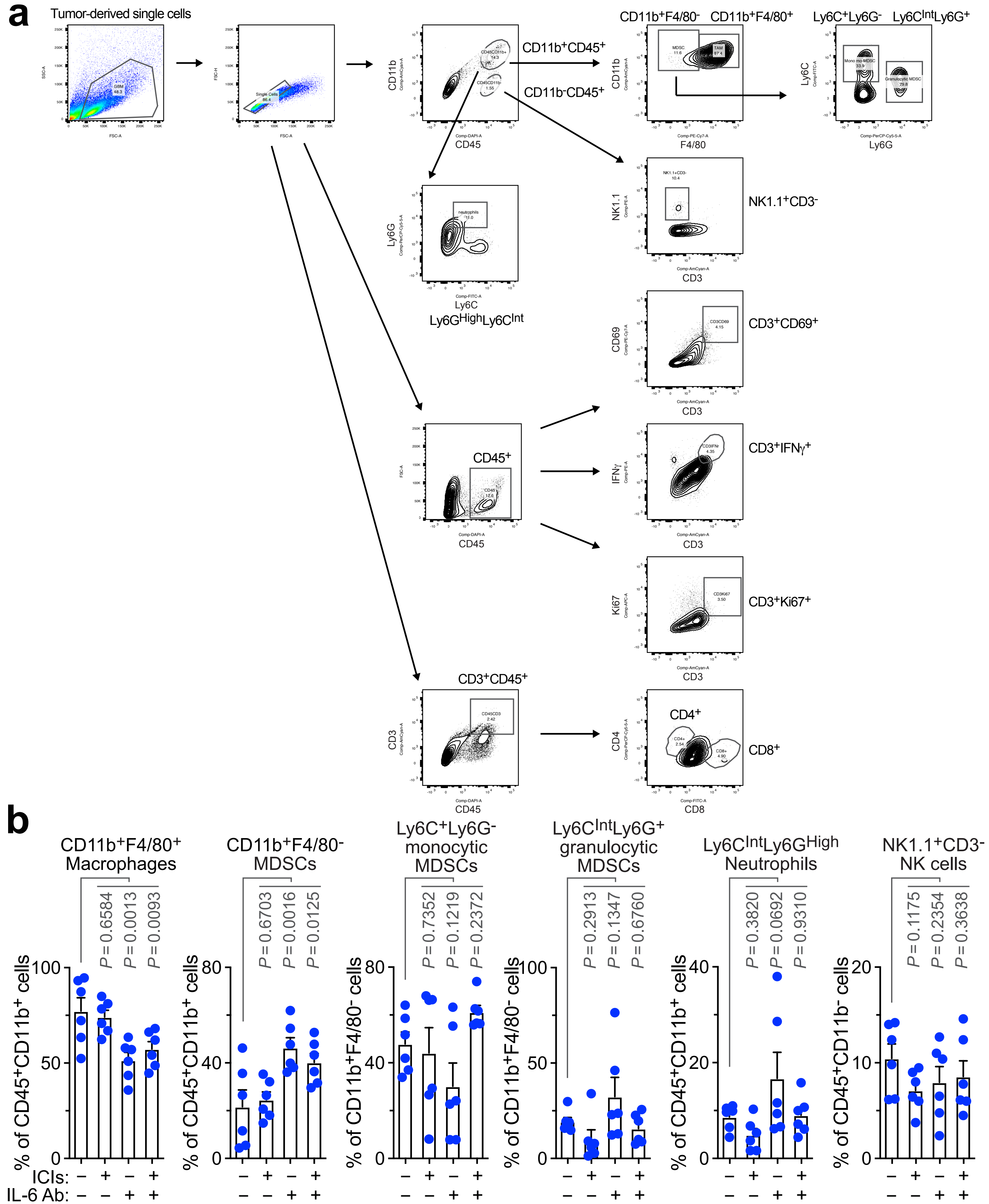

Supplementary Fig. 2. Effects of IL-6 blockade and immune checkpoint inhibition on GBM immunity. GBM was induced in WT mice, followed by injection with control IgG, anti-IL-6 Ab, and/or immune checkpoint inhibition (ICIs). Tumors were excised 3 days after treatment. Tumor-derived single cell suspensions were analyzed by flow cytometry. **a**, Gating strategies used for the assays presented in this figure and Fig. 2d-g. **b**, Analysis for different immune cells (n = 6 mice, mean ± SEM). Statistical analysis by one-way ANOVA with Fisher's LSD test. Source data are provided as a Source data file.

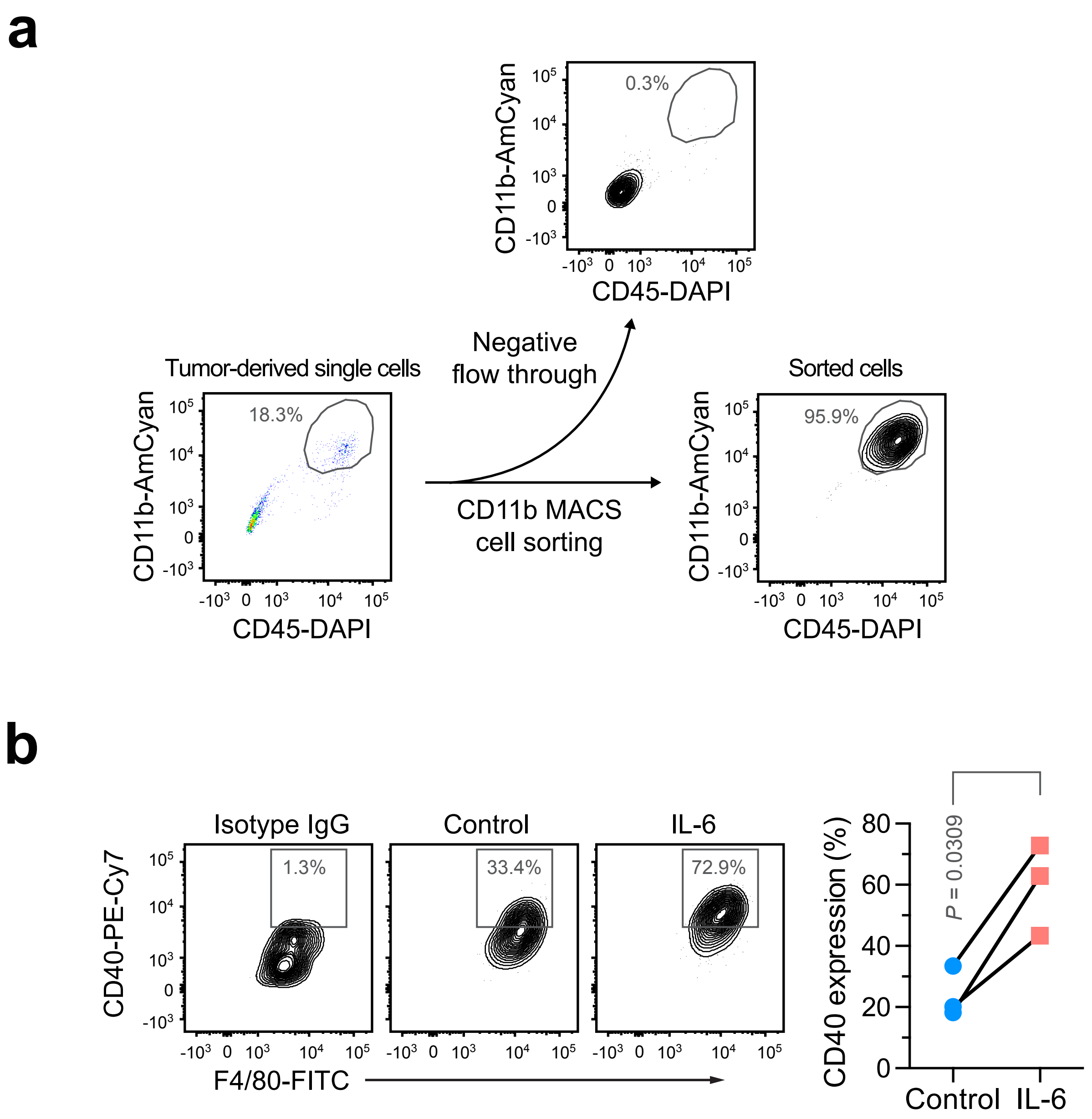

Supplementary Fig. 3. IL-6 stimulates CD40 expression in tumor-derived Mϕs.
GBM was genetically induced in WT mice. Tumor-derived single cell suspensions were sorted by magnetic activated cell sorting (MACS) with anti-CD11b-conjugated beads. **a**, Cells before and after MACS were analyzed by flow cytometry with anti-CD11b and anti-CD45 antibodies. Representative cell sortings are shown (n = 3 mice). **b**, Sorted $CD11b^+$ cells were treated with 50 ng/ml IL-6 for 2 days, followed by flow cytometry analysis. Left, representative cell sortings. Right, quantified results (n = 3 mice). Statistical analysis by two-tailed paired Student's *t* test. Source data are provided as a Source data file.

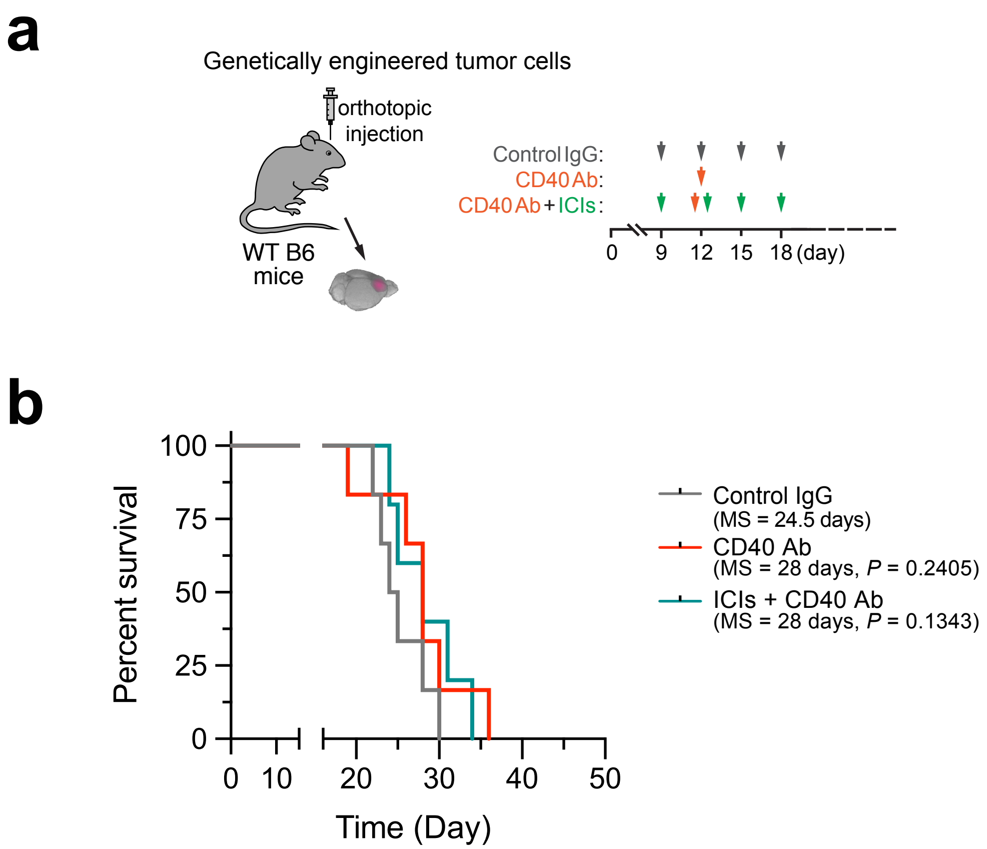

Supplementary Figure 4. Combination therapy with a CD40 agonist and checkpoint inhibitors does not extend animal survival in GBM-bearing mice.
GBM was induced in mice, followed by different treatment and survival analyses (n = 6 mice for control IgG group and CD40 Ab group, and n = 5 mice for ICIs plus CD40 Ab group). (**a**) Experimental procedure. (**b**) Mouse survival was monitored and analyzed by Log-rank Mantel-cox analysis.

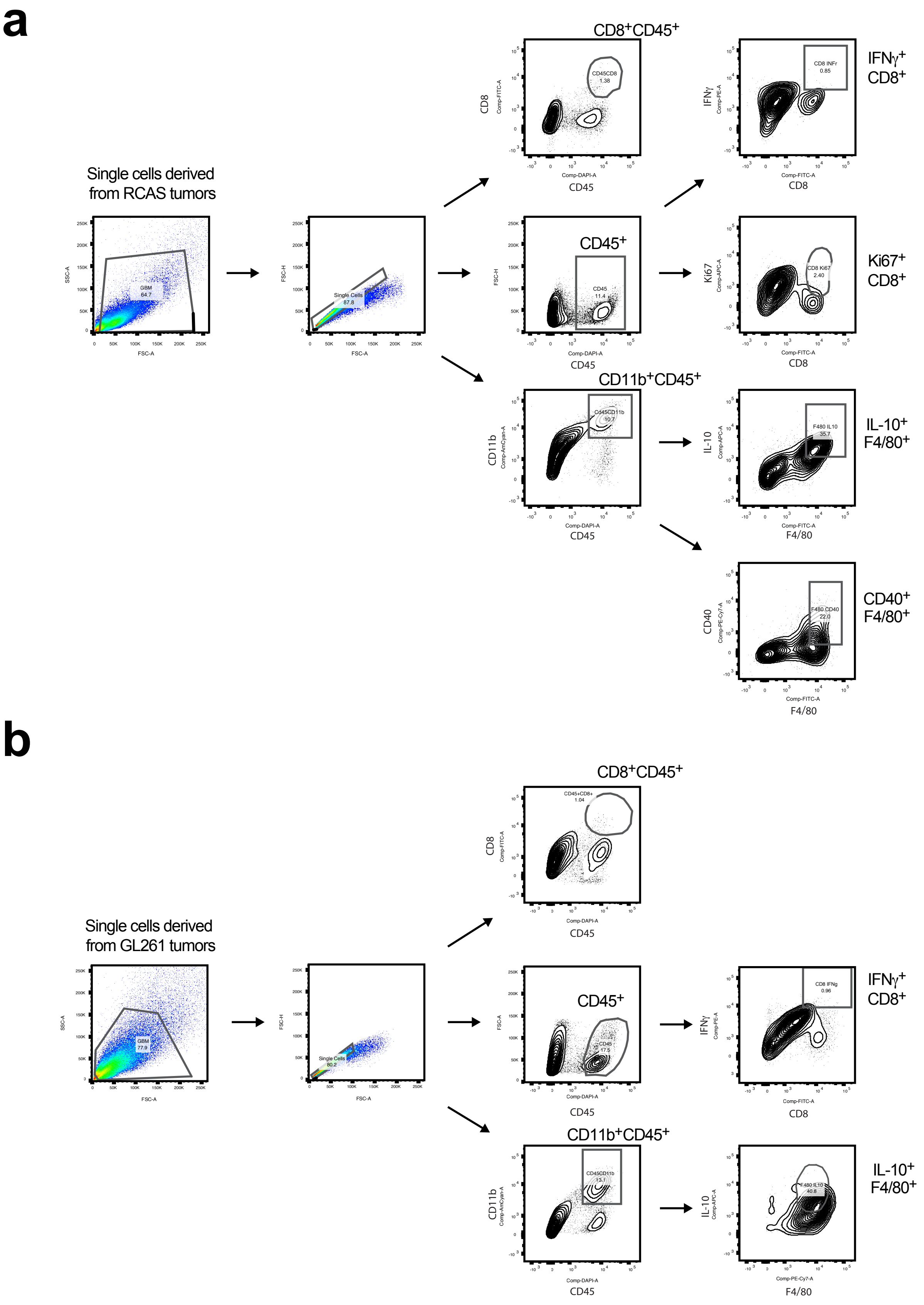

Supplementary Fig. 5. Gating strategies for flow cytometry analysis of mouse GBM tumors treated with ICIs, IL-6 antibody, and CD40 antibody.

GBM was induced in mice by injection with **a**, tumor cells derived from RCAS-mediated genetically eningeered GBM model or with **b**, GL261 mouse glioma cells, followed by treatment with control antibody, ICIs, IL-6 antibody, and CD40 antibody. Tumor-derived single cells were stained with different antibodies and analyzed by flow cytometry. **a**, Gating strategies used for the assays presented in Fig. 3i,j and Fig. 6c-f. **b**, Gating strategies used for the assays presented in Supplementary Fig. 6b-d.

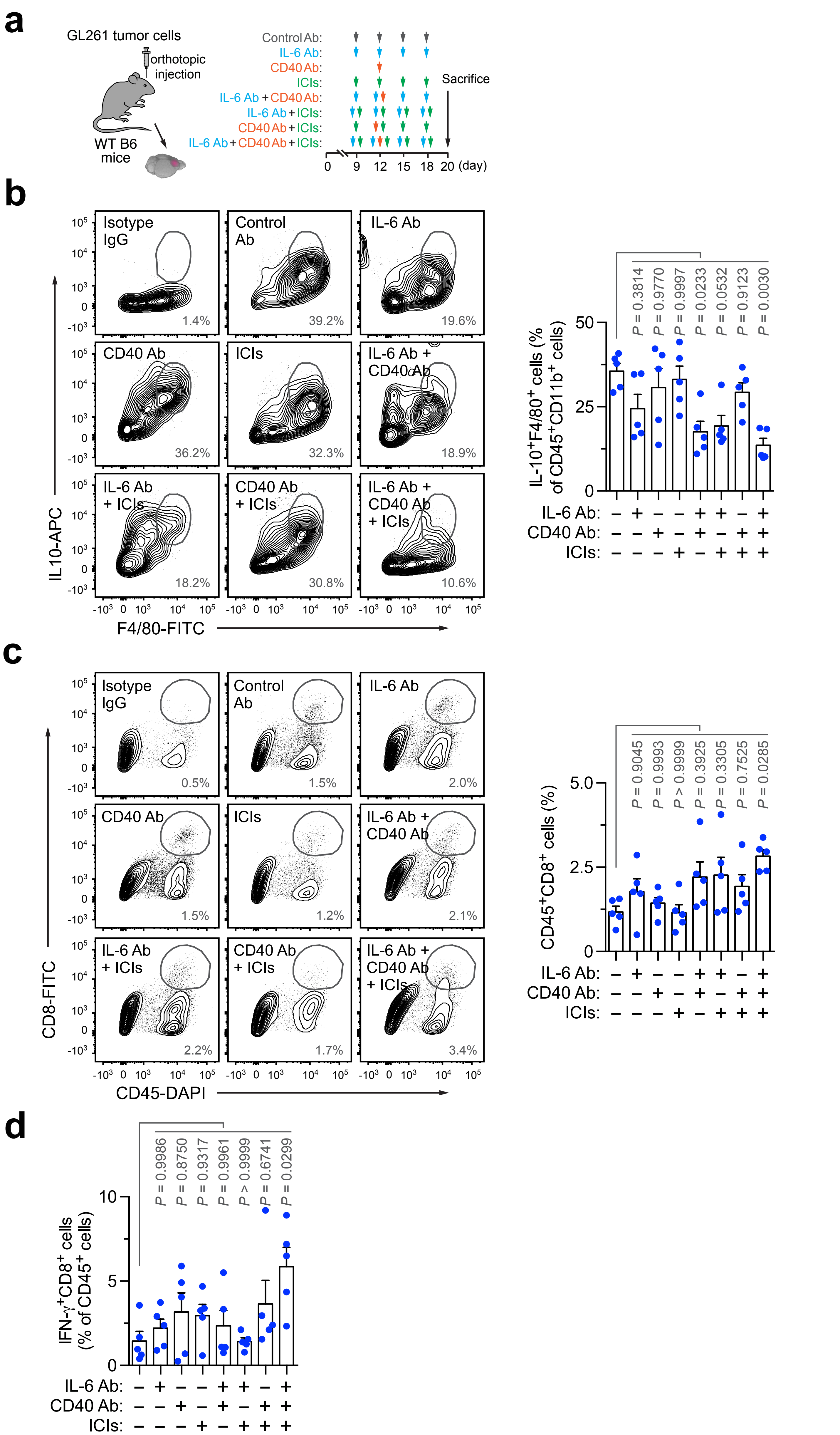

Supplementary Fig. 6. IL-6 neutralization and CD40 stimulation plus immune checkpoint blockade synergistically reverses Mϕ-mediated immune suppression and activates tumor-associated T cells in GL261 GBM.
GBM was induced in mice by injection with GL261 mouse glioma cells, followed by different treatment and end-point analyses. **a**, Experimental procedure. **b**-**e**, Tumor-derived single-cell suspensions were analyzed by flow cytometry. **b**,**c**, Cells were probed with **b**, anti-F4/80 and anti-IL10 or **c**, anti-CD45 and anti-CD8 antibodies. Left, representative sortings. Right, quantified results (n = 5 mice, mean ± SEM). Statistical analysis by one-way ANOVA with Turkey's test. **d**, Cells were probed with anti-CD8 and anti-IFN-γ antibodies. Quantified results are shown (n = 5 mice, mean ± SEM). Statistical analysis by one-way ANOVA with Turkey's test. Source data are provided as a Source data file.

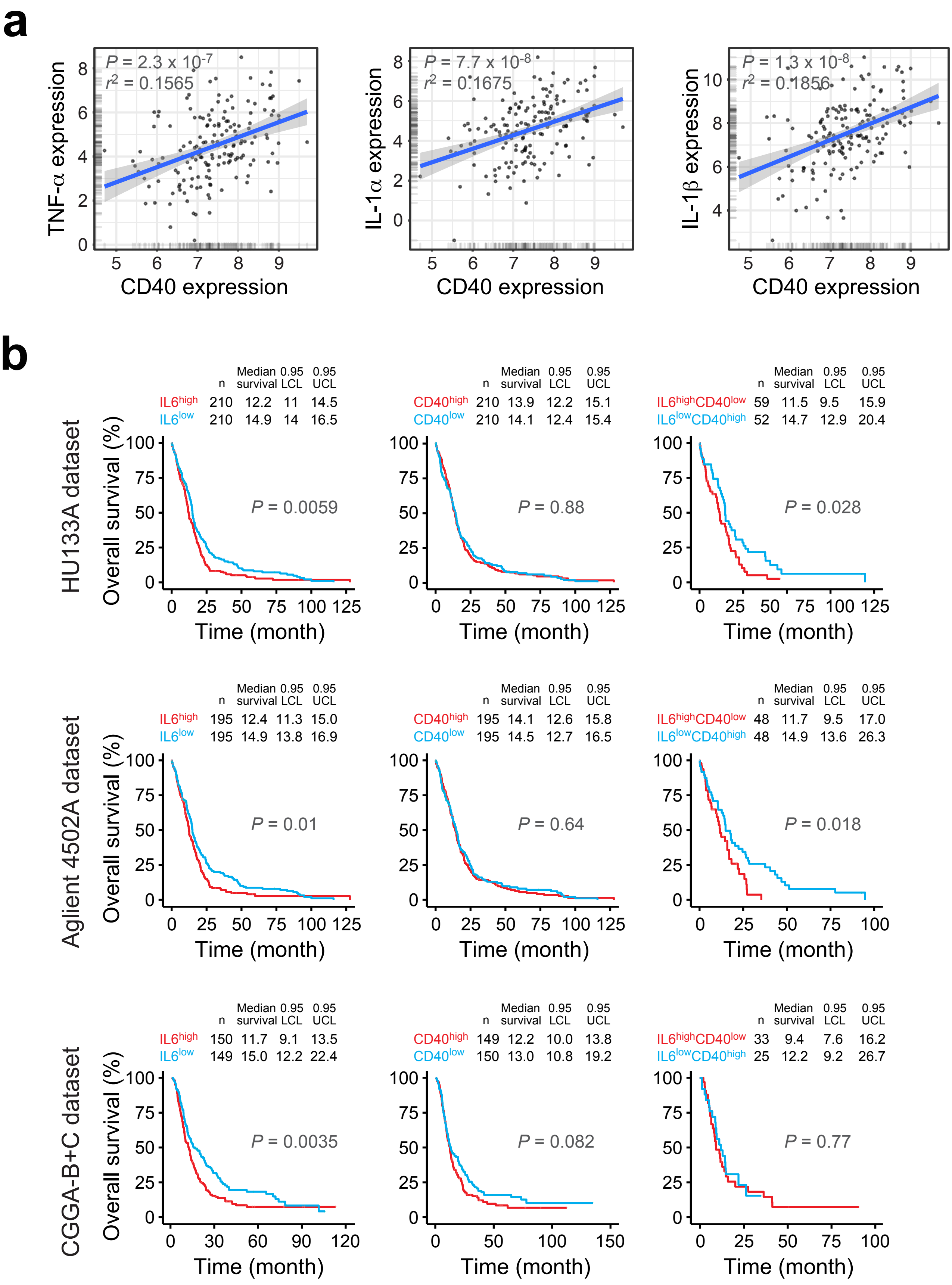

Supplementary Fig. 7. High IL-6 expression and low CD40 expression correlate with poor survival in human GBM patients. **a**, Correlation of CD40 expression with TNF-α, IL-1a, and IL-1b was subjected to linear regression analyses using GlioVis/TCGA GBM-RNAseq dataset (n = 160 patients). Statistical analysis by linear regression analysis. **b**, Correlation of IL6 and CD40 expression (high/low cutoff of 40%) with overall survival was analyzed using GlioVis datasets. Statistical analysis by two-sided Log-rank test.
