# Supplementary material for: Synergistic immunotherapy of glioblastoma by dual targeting of IL-6 and CD40
Source: Nat Commun. 2021 Jun 8;12:3424. doi: 10.1038/s41467-021-23832-3 (PMC8187342; doi:10.1038/s41467-021-23832-3)
Supplement: Supplementary file 2 — Reporting Summary [file 41467_2021_23832_MOESM2_ESM.pdf]

nature research

Corresponding author(s): Yi Fan

Last updated by author(s): May 6, 2021

Reporting Summary

Nature Research wishes to improve the reproducibility of the work that we publish. This form provides structure for consistency and transparency in reporting. For further information on Nature Research policies, see our Editorial Policies and the Editorial Policy Checklist.

Statistics

For all statistical analyses, confirm that the following items are present in the figure legend, table legend, main text, or Methods section.

n/a	Confirmed	
☐	☒	The exact sample size (*n*) for each experimental group/condition, given as a discrete number and unit of measurement
☐	☒	A statement on whether measurements were taken from distinct samples or whether the same sample was measured repeatedly
☐	☒	The statistical test(s) used AND whether they are one- or two-sided<br>*Only common tests should be described solely by name; describe more complex techniques in the Methods section.*
☒	☐	A description of all covariates tested
☐	☒	A description of any assumptions or corrections, such as tests of normality and adjustment for multiple comparisons
☒	☐	A full description of the statistical parameters including central tendency (e.g. means) or other basic estimates (e.g. regression coefficient) AND variation (e.g. standard deviation) or associated estimates of uncertainty (e.g. confidence intervals)
☐	☒	For null hypothesis testing, the test statistic (e.g. *F*, *t*, *r*) with confidence intervals, effect sizes, degrees of freedom and *P* value noted<br>*Give P values as exact values whenever suitable.*
☒	☐	For Bayesian analysis, information on the choice of priors and Markov chain Monte Carlo settings
☒	☐	For hierarchical and complex designs, identification of the appropriate level for tests and full reporting of outcomes
☒	☐	Estimates of effect sizes (e.g. Cohen's *d*, Pearson's *r*), indicating how they were calculated

Our web collection on statistics for biologists contains articles on many of the points above.

Software and code

Policy information about availability of computer code

Data collection	Illumina sequencer HiSeq 2500 for RNAseq data.
Data analysis	FlowJo v9 for flow cytometry data.<br>R software (Version 3.6.3) for dataset analysis.<br>Cytobank software (7.3.0) for CyTOF analysis.<br>RNA-Star (v2.4.2a) and Cufflinks (v2.2.1) for RNAseq analysis.<br>Graphpad Prism 8 for statistical analysis.

For manuscripts utilizing custom algorithms or software that are central to the research but not yet described in published literature, software must be made available to editors and reviewers. We strongly encourage code deposition in a community repository (e.g. GitHub). See the Nature Research guidelines for submitting code & software for further information.

Data

Policy information about availability of data

All manuscripts must include a data availability statement. This statement should provide the following information, where applicable:

- Accession codes, unique identifiers, or web links for publicly available datasets
- A list of figures that have associated raw data
- A description of any restrictions on data availability

RNA-seq data have been deposited in NCBI's Gene Expression Omnibus (GSE151213). Source data are available as a Source data file. Gene expression and survival data of glioma patients were obtained from TCGA (https://portal.gdc.cancer.gov) and GlioVis (http://gliovis.bioinfo.cnio.es/). The remaining data are available within the Article and Supplementary Information or available from the authors upon request.

Field-specific reporting

Please select the one below that is the best fit for your research. If you are not sure, read the appropriate sections before making your selection.

- [x] Life sciences
- [ ] Behavioural & social sciences
- [ ] Ecological, evolutionary & environmental sciences

For a reference copy of the document with all sections, see nature.com/documents/nr-reporting-summary-flat.pdf

Life sciences study design

All studies must disclose on these points even when the disclosure is negative.

Sample size	The sample size was chosen in advance based on common practice of the described experiments in the literature and our previous work (PMID: 32102932 and 27043280). Sample sizes for comparisons also followed Mead's recommendations. At least 3 of samples per group for all in vitro and in vivo experiments.
Data exclusions	No data excluded.
Replication	Technical replicates and independent experiments were performed to verify reproducibility of the assays. The replication information is provided in the figure legends. All attempts at replication were successful.
Randomization	In cell culture experiments, cell dishes were randomly assigned to different study group. In animal studies, all mice used were age- and gender-matched, and were randomly selected.
Blinding	Blinding was not carried out in cell culture experiments or in data collection, since all experiments had to be carried out by the same researchers. In animal studies, investigators were not blinded, as all mouse treatment and tissue analysis experiments had to be performed by the same researchers.

Reporting for specific materials, systems and methods

We require information from authors about some types of materials, experimental systems and methods used in many studies. Here, indicate whether each material, system or method listed is relevant to your study. If you are not sure if a list item applies to your research, read the appropriate section before selecting a response.

Materials & experimental systems

n/a	Involved in the study	
[ ]	[x]	Antibodies
[ ]	[x]	Eukaryotic cell lines
[x]	[ ]	Palaeontology and archaeology
[ ]	[x]	Animals and other organisms
[ ]	[x]	Human research participants
[x]	[ ]	Clinical data
[x]	[ ]	Dual use research of concern

Methods

n/a	Involved in the study	
[x]	[ ]	ChIP-seq
[ ]	[x]	Flow cytometry
[x]	[ ]	MRI-based neuroimaging

Antibodies

Antibodies used

anti-rabbit IgG (1:100, Santa Cruz, sc-2027, Polyclonal) for ChIP
anti-rabbit or anti-mouse IgG-HRP conjugate (1:5,000, Bio-Rad, 1706515 or 1706516) for immunoblot
anti-CD3 (1:100, BioLegend,100233, 17A2 Monoclonal) for flow cytometry
anti-CD8a (1:100, BioLegend, 100706, 53-6.7 Monoclonal) for flow cytometry
anti-CD4 (1:100, BioLegend, 100540, RM4-5 Monoclonal) for flow cytometry
anti-CD69 (1:200, BioLegend, 104511, H1.2F3 Monoclonal) for flow cytometry
anti-IFN-g (1:100, BioLegend, 505808, XMG1.2 Monoclonal) for flow cytometry
anti-Ki67 (1:100, BioLegend, 652405, 16A8 Monoclonal) for flow cytometry
anti-CD45 (1:200, eBioscience, 48-0451-82, 30-F11 Monoclonal) for flow cytometry
anti-CD11b (1:200, BioLegend, 101228, M1/70 Monoclonal) for flow cytometry
anti-CD11b (1:200, eBioscience, 69-0112-80, M1/70 Monoclonal) for flow cytometry
anti-CD11c (1:200, eBioscience, 17-0114-81, N418 Monoclonal) for flow cytometry
anti-F4/80 (1:200, BioLegend, 123107, BM8 Monoclonal) for flow cytometry
anti-CD206 (1:100, BioLegend, 141710, C068C2 Monoclonal) for flow cytometry
anti-Ly6G (1:200, BioLegend, 127615, 1A8 Monoclonal) for flow cytometry
anti-Ly6C (1:200, BioLegend, 128005, HK1.4 Monoclonal) for flow cytometry
anti-IL10 (1:100, BioLegend, 505009, JES5-16E3 Monoclonal) for flow cytometry
anti-Gr1 (1:200, BioLegend, 108415, RB6-8C5 Monoclonal) for flow cytometry

anti-MHCII (1:100, eBioscience, 47-5321-80, M5/114.15.2 Monoclonal) for flow cytometry
anti-CD40 (1:100, BioLegend, 124621/124611, 3/23 Monoclonal) for flow cytometry
anti-NK1.1 (1:100, BioLegend, 108707, PK136 Monoclonal) for flow cytometry
anti-B220 (1:100, eBioscience, 47-0452-80, RA3-6B2 Monoclonal) for flow cytometry
anti-HIF-1α (1:1000 or 1:50, CST, 14179, D2U3T Monoclonal) for immunoblot or ChIP
anti-Stat3 (1:1000 or 1:50, CST, 12640, D3Z2G Monoclonal) for immunoblot or ChIP
anti-NF-kB (1:1000, CST, 4882, Polyclonal) for immunoblot
anti-CD40 (1:1000, CST, 86165, E2Z7J Monoclonal) for immunoblot
anti-GAPDH (1:3000, CST, 5174, D16H11 Monoclonal) for immunoblot

Validation

All antibodies were purchased from commercial sources and have been validated by the vendors. Additional validation has also been given in previous publication with PubMed IDs listed:
anti-rabbit IgG (Santa Cruz, sc-2027) PMID: 11285237, 31744881, 21315255
anti-rabbit or anti-mouse IgG-HRP conjugate (Bio-Rad, 1706515 or 1706516) PMID: 31063986, 30081710, 31038471
anti-CD3 (BioLegend,100233) PMID: 28560793, 29456159,30393066
anti-CD8a (BioLegend, 100706) PMID: 29129787, 29363160, 29777108
anti-CD4 (BioLegend, 100540) PMID: 29429633, 30446387, 30076101
anti-CD69 (BioLegend, 104511) PMID: 28700944, 29669249, 30540939
anti-IFN-g (BioLegend, 505808) PMID: 29136509, 29491374, 29160310
anti-Ki67 (BioLegend, 652405) PMID: 30005826, 29958803, 28844880
anti-CD45 (eBioscience, 48-0451-82) PMID: 31481662, 31408442, 31018138
anti-CD11b (BioLegend, 101228) PMID: 28939717, 29867149, 30170810
anti-CD11b (eBioscience, 69-0112-80) PMID: 31481966, 31780645, 30970257
anti-CD11c (eBioscience, 17-0114-81) PMID: 30333482, 29972778, 29504948
anti-F4/80 (BioLegend, 123107) PMID: 28939843, 29664018, 29070674
anti-CD206 (BioLegend, 141710) PMID: 31409776, 30770245, 30898010
anti-Ly6G (BioLegend, 127615) PMID: 28592427, 29414687, 30220458
anti-Ly6C (BioLegend, 128005) PMID: 30389414, 30318149, 30733433
anti-IL10 (BioLegend, 505009) PMID: 29422647, 30540937, 30926232
anti-Gr1 (BioLegend, 108415) PMID: 29456159, 30423295, 30301799
anti-MHCII (eBioscience, 47-5321-80) PMID: 22919031, 31914398, 29354128
anti-CD40 (BioLegend, 124621) PMID: 31015515, 31801074, 26303108
anti-NK1.1 (BioLegend, 108707) PMID: 29070816, 29255233, 29403003
anti-B220 (eBioscience, 47-0452-80) PMID: 30687320, 31230859, 29925839
anti-HIF-1α (CST, 14179) PMID: 15545609, 12149254, 11292861
anti-Stat3 (CST, 12640) PMID: 9108058, 8197455, 10023775
anti-NF-kB (CST, 4882) PMID: 8858144, 1829648, 7867065
anti-CD40 (CST, 86165) PMID: 23892087, 12576427, 19426221
anti-GAPDH (CST, 5174) PMID: 12887926, 16492755, 15746184

Eukaryotic cell lines

Policy information about cell lines

Cell line source(s)	DF-1 cells were obtained from ATCC, and GL261 cells were from PerkinElmer.
Authentication	Cell lines were authenticated by the supplying institute or company. Cell lines were not authenticated by the investigators.
Mycoplasma contamination	All cell lines have been tested and shown negative for mycoplasma contamination.
Commonly misidentified lines (See ICLAC register)	There were no commonly misidentified cell lines used in this study.

Animals and other organisms

Policy information about studies involving animals; ARRIVE guidelines recommended for reporting animal research

Laboratory animals	Wild-type (WT) mice on the C57BL/6J background were obtained from Jackson Lab. Cdh5-CreERT2;Il6fl/fl mice were generated by crossing Il6fl/fl mice with Cdh5-CreERT2 mice. All animals were housed at room temperature with a 12-light/12-dark cycle in the Association for the Assessment and Accreditation of Laboratory Animal Care-accredited animal facility of the University of Pennsylvania. For tumor induction experiments, both female and male eight-week-old mice were used.
Wild animals	The study did not involve wild animals.
Field-collected samples	The study did not involve samples collected from the field.
Ethics oversight	All experiments with mice were performed in accordance with a protocol approved by the Institutional Animal Care and Use Committee (IACUC) at the University of Pennsylvania.

Note that full information on the approval of the study protocol must also be provided in the manuscript.

Human research participants

Policy information about studies involving human research participants

Population characteristics	Healthy adult human volunteer donors with ages 18 to 65 and with all genders, races, and ethnicities.
Recruitment	Primary human monocytes were harvested and provided by Human Immunology Core at the University of Pennsylvania. Peripheral blood mononuclear cells (PBMC) were harvested from collected blood of healthy human volunteer donors.
Ethics oversight	All specimens were collected under a University of Pennsylvania Institutional Review Board-approved protocol and written informed consent was obtained from each donor.

Note that full information on the approval of the study protocol must also be provided in the manuscript.

Flow Cytometry

Plots

Confirm that:

- [x] The axis labels state the marker and fluorochrome used (e.g. CD4-FITC).
- [x] The axis scales are clearly visible. Include numbers along axes only for bottom left plot of group (a 'group' is an analysis of identical markers).
- [x] All plots are contour plots with outliers or pseudocolor plots.
- [x] A numerical value for number of cells or percentage (with statistics) is provided.

Methodology

Sample preparation	Tumors were isolated and subjected to mechanical dissociation with a gentleMACS Dissociator (Miltenyi) and enzymatic digestion with collagenase II and dispase II to obtain single cell suspensions. Macrophages were isolated from mouse bone marrow, followed by different treatments. Single-cell suspensions were stained with control isotype IgG or fluoresence-conjugated antibody, followed by flow cytometry analysis.
Instrument	Accuri C6 and Canto II (BD Biosciences)
Software	FlowJo software
Cell population abundance	More than 200 thousand cells were sorted.
Gating strategy	All cells were gated.

- [x] Tick this box to confirm that a figure exemplifying the gating strategy is provided in the Supplementary Information.
